# Laboratory observation of ion drift acceleration via reflection off laser-produced magnetized collisionless shocks

**DOI:** 10.1126/sciadv.adn3320

**Published:** 2025-02-12

**Authors:** Hui-bo Tang, Yu-fei Hao, Guang-yue Hu, Quan-ming Lu, Chuang Ren, Yu Zhang, Ao Guo, Peng Hu, Yu-lin Wang, Xiang-bing Wang, Zhen-chi Zhang, Peng Yuan, Wei Liu, Hua-chong Si, Chun-kai Yu, Jia-yi Zhao, Jin-can Wang, Zhe Zhang, Xiao-hui Yuan, Da-wei Yuan, Zhi-yong Xie, Jun Xiong, Zhi-heng Fang, Jian-cai Xu, Jing-Jing Ju, Guo-qiang Zhang, Jian-Qiang Zhu, Ru-xin Li, Zhi-zhan Xu

**Affiliations:** ^1^ CAS Key Laboratory of Geospace Environment, University of Science and Technology of China, Hefei, China.; ^2^ School of Earth and Space Sciences, University of Science and Technology of China, Hefei, China.; ^3^ School of Physics, Harbin Institute of Technology, Harbin, China.; ^4^ Key Laboratory of Planetary Sciences, Purple Mountain Observatory, Chinese Academy of Sciences, Nanjing, China.; ^5^ CAS Center for Excellence in Comparative Planetology, Hefei, China.; ^6^ School of Nuclear Science and Technology & School of Physical Science, University of Science and Technology of China, Hefei, China.; ^7^ State Key Laboratory of High Field Laser Physics & CAS Center for Excellence in Ultra-intense Laser Science, Shanghai Institute of Optics and Fine Mechanics, Chinese Academy of Sciences, Shanghai, China.; ^8^ Department of Mechanical Engineering, University of Rochester, Rochester, NY, USA.; ^9^ Institute of Physics, Chinese Academy of Sciences, Beijing, China.; ^10^ Key Laboratory for Laser Plasmas (Ministry of Education), School of Physics and Astronomy, Shanghai Jiao Tong University, Shanghai, China.; ^11^ Key Laboratory of Optical Astronomy, National Astronomical Observatories, Chinese Academy of Sciences, Beijing, China.; ^12^ Shanghai Institute of Laser Plasma, Shanghai, China.; ^13^ Shanghai Institute of Applied Physics, Chinese Academy of Sciences, Shanghai, China.; ^14^ National Laboratory on High Power Laser and Physics, Shanghai Institute of Optics and Fine Mechanics, Chinese Academy of Sciences, Shanghai, China.

## Abstract

Fermi acceleration is believed to be the primary mechanism to produce high-energy charged particles in the Universe, where charged particles gain energy successively from multiple reflections. Here, we present the direct laboratory experimental evidence of ion energization from single reflection off a supercritical collisionless shock, an essential component of Fermi acceleration, in a laser-produced magnetized plasma. A quasi-monoenergetic ion beam with two to four times the shock velocity was observed, which is consistent with the fast ion component observed in the Earth’s bow shock. Our simulations reproduced the energy gain and showed that ions were accelerated mainly by the motional electric field during reflection. The results identify shock drift acceleration as the dominant ion energization mechanism, which is consistent with satellite observation in the Earth’s bow shock. Our observations pave the way for laboratory investigations of the cosmic accelerators, also be beneficial to laser fusion and laser-driven ion accelerator.

## Teaser

A quasi-monoenergetic ion beam was observed in laser-produced magnetized collisionless shock via ion drift acceleration.

## INTRODUCTION

Collisionless shocks are among the most powerful particle accelerators in astrophysics (*1*, *2*). They act as the moving scattering centers, originally proposed by Fermi as an origin of cosmic rays (*3*), where charged particles gain energy by reflecting off them. A succession of small energy increments due to repeated shock crossings back and forth between the upstream and downstream creates the power law spectrum of energetic particles, a process known as diffusive shock acceleration (*1*, *4*–*7*). To enter the Fermi energization cycle, particles must be preaccelerated to have a gyroradius large enough to be able to scatter between upstream and downstream. Several competing mechanisms including shock drift acceleration (SDA) and shock surfing acceleration (SSA) have been proposed to solve this well-known “injection problem” (*8*–*10*), all in theory or simulations (*11*–*24*).

In situ spacecraft measurements have addressed the fundamental question of collisionless shock physics, although it remains fundamentally limited due to undersampling (*25*, *26*). As a result, the formation and evolution of collisionless shocks are not fully understood. Laboratory experiments (*27*–*37*) with controllable and reproducible conditions can complement some of these limitations and have recently extended to supercritical magnetized collisionless shock related to the Solar System (*38*–*40*). At the Omega laser facility, the formation (*38*) and particle dynamics (*39*) of high–Mach number magnetized collisionless shock (magnetosonic Mach number *M_ms_* ~ 12) were measured in situ via an optical and proton probe beam. Experiments at the LULI laser facility observed weak continuous ion spectrum produced by low–Mach number shock (*M_ms_* ~ 3.1), which was attributed to SSA mechanisms (*41*). Even so, the effectiveness and relative importance of SDA and SSA still remain unanswered (*41*–*46*).

Here, we report on experimental results of ion acceleration in a middle–Mach number (*M_ms_* ~ 6) supercritical quasi-perpendicular collisionless shock formed when a laser-produced supersonic plasma flow impact on a magnetized ambient plasma. Quasi-monoenergetic ions with two to four times the shock velocity are observed in the upstream of shock, and the energetic ion flux is three to four orders higher than that in previous experiment of SSA (*40*). It is the direct laboratory experimental evidence of ion acceleration from single reflection off a collisionless shock, which is in well agreement with the fast ion component observed in the Earth’s bow shock (*47*–*51*). Our results indicate that SDA dominates the ion energization in the Earth’s bow shock (*41*–*45*, *48*, *51*), not SSA claimed previously (*40*). Ions are accelerated along both the shock normal and the shock front and mainly by the motional electric field during reflection from the shock.

## RESULTS

**Fig. 1.**
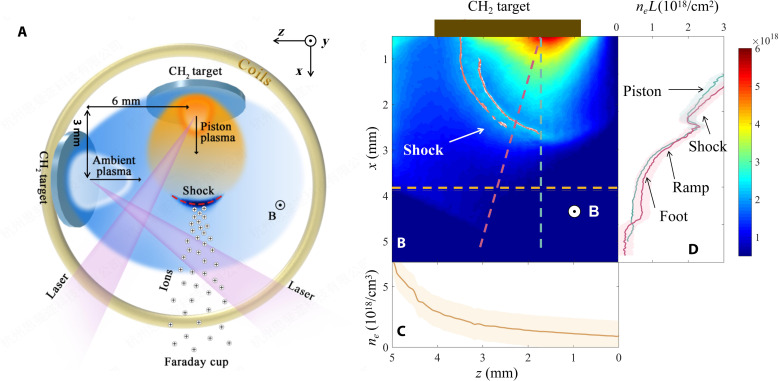
Laser-driven magnetized collisionless shock experiments. (**A**) Sketch of the experimental setup: A 4- to 6-T external magnetic field (along the *y* direction) was applied by a pulsing current through a set of magnetic field coils. Ambient plasma was generated after the plastic CH_2_ target (left) was ablated by a weaker precursor beam (100 J/1 ns/351 nm). After 12 ns (at time *t*_0_) where the ambient plasma was magnetized via an anomalous magnetic diffusion process, an intense drive beam (260 J/1 ns/351 nm) irradiated another plastic CH_2_ target (top) to produce a supersonic piston plasma flow, which drove the collisionless shock in the magnetized ambient plasma. The density profiles of the shock and the ambient plasma were characterized with optical diagnostics along the *y* axis. The acceleration of ions was measured by the TOF method using a Faraday cup (directed along the *x* axis). (**B**) Imaging of shock measured by optical interferometry and the dark-field schlieren method (red contours) (line integrated along the *y* direction), taken at time *t*_0_+4 ns, formed in the ambient plasma with a 5-T external magnetic field. The inhomogeneous ambient plasma results in an asymmetric quasi-hemispherical shock. (**C**) Electron density profile for the ambient plasma, taken at time *t*_0_+4 ns along the yellow line in (B) at *x* = 4 mm, under the experimental condition without a piston plasma flow, which varies from *n*_e0_ ~ 1 × 10^18^/cm^3^ to 5 × 10^18^/cm^3^ with a gradient scale length of ~1 mm in the shock traveling zone. (**D**) Line-integrated electron density profile of shock taken along the red line in (B) with (red) and without (gray) external magnetic field. *L* is the plasma size in the *y* direction. The electron densities in upstream and downstream are ~1 × 10^18^/cm^3^ to 5 × 10^18^/cm^3^ and 0.5 × 10^19^/cm^3^ to 1.5 × 10^19^/cm^3^ (see details in fig. S3), respectively, which indicate a compression ratio of >3.

The experiments were conducted at the Shenguang-**II** laser facility. A sketch of the experimental setup is shown in Fig. 1A. A weaker precursor laser beam (~1 × 10^13^ W/cm^2^) ablated a plastic (CH_2_) planar target to create the ambient plasma, which was magnetized by a 5- to 6-T external background magnetic field (*52*) via an anomalously fast magnetic diffusion process (*39*, *53*–*55*). An intense drive laser beam (~8 × 10^13^ W/cm^2^) irradiated another plastic (CH_2_) target with a focus spot of 0.5 by 0.5 mm^2^ to produce supersonic plasma flow as a piston. The piston plasma flow drove a quasi-perpendicular collisionless shock in the magnetized ambient plasma. The profile of the shock and the ambient plasma density were characterized with optical diagnostics. The ion velocity spectrum was measured by the time-of-flight (TOF) method using a Faraday cup (see Materials and Methods for further details).

The electron density of the ambient plasma varies from ~1 × 10^18^/cm^3^ to 5 × 10^18^/cm^3^ with a gradient scale length of ~1 mm (Fig. 1C), and the electron temperature is estimated to be ~40 ± 10 eV (*39*, *56*). The piston plasma with a higher electron temperature of ~200 eV (*39*, *56*) can drive a quasi-hemispherical magnetized collisionless shock (Fig. 1B), which is asymmetric due to the inhomogeneity of the ambient plasma (Fig. 1C). A strongly compressed zone is formed within the plasma, and the plasma density exhibits a typical shock structure of a “foot,” a “ramp,” etc. (Fig. 1D and figs. S3 and S14) (*37*–*40*, *56*, *57*). The narrower schlieren band with an external magnetic field (Fig. 1D) indicates that the magnetic field contributes to the formation of the shock compared to that without a magnetic field, which is coincident with the previous measurement of the magnetic field topology (*39*, *56*). When the magnetic field is applied, another noticeable feature, as observed by satellites crossing the Earth’s bow shock and in previous simulations (*41*–*45*), is the broader and denser foot region in the density profile (Fig. 1D) caused by reflected ions. These features can indicate the formation of a magnetized shock. The angle between the shock normal and the upstream magnetic field θ*_Bn_* in our experiments is ~90°; therefore, it is a nearly perpendicular shock (see Materials and Methods for further details). The shock velocity is *v*_shock_ ~ 400 km/s over the span of measurement, which is slightly slower than that without an external magnetic field (fig. S4) yet still within the measurement error.

Under our experimental parameters, the magnetized shock is approximately collisionless. The ion-ion collisional mean free path is ~4 mm, which is much larger than the ion Larmor radius of ~800 μm and the shock thickness of ~500 μm. The >3× density compression factor approximately satisfies the hydrodynamic Rankine-Hugoniot jump condition of shock (*57*). The shock Alfvénic, sonic, and magnetosonic Mach numbers are *M_A_* ~ 7 to 11, *M_s_* ~ 7 to 9, and *M_ms_* ~ 5 to 7, respectively, and the ambient plasma beta value is β ~ 0.9 to 1.4. Therefore, the shock conditions probed in our experiments are relevant to the Earth’s bow shock, where the typical shock Alfvénic Mach number is *M_A_* ~ 3 to 10 (*48*, *49*, *58*–*61*), as illustrated in Table 1.

**Table 1.** Parameters of collisionless shock in laboratory and astrophysical environments.

Parameters	Our exp.	Our sim.	Bow shock (*47*–*49*, *59*, *60*)	Term. shock (*76*)	SNR (SN1006) (*34*)
Flow velocity (km/s)	400 to 500	400	400	300	3000 to 5000
*B* (G)	(5 to 6) × 10^4^	6 × 10^4^	6 × 10^−5^	1 × 10^−6^	3 × 10^−6^
Electron temperature (eV)	40 ± 10	60	15	1	1
Sound velocity *c*_s_ (km/s)	57	40	50	13	13
Alfvénic velocity (km/s)	40 to 60	50	50	49	15
Ion thermal velocity (km/s)	140	22	50	10	10
Collisional mean free path λ_mfp_ (cm)	0.4		1 × 10^16^	1.3 × 10^19^	3 × 10^21^
Ion Larmor radius *r*_ci_ (cm)	0.08		7 × 10^6^	1 × 10^8^	3.4 × 10^7^
λ_mfp_/*r*_ci_	5		2 × 10^9^	1 × 10^11^	1 × 10^14^
Beta	0.9 to 1.4	1.005	1.2	0.081	0.9
M_s_	7 to 9	10.4	5 to 10	24	200 to 400
M_A_	7 to 11	8.3	3 to 10	6	200 to 400
M_ms_	5 to 7	6.5			

**Fig. 2.**
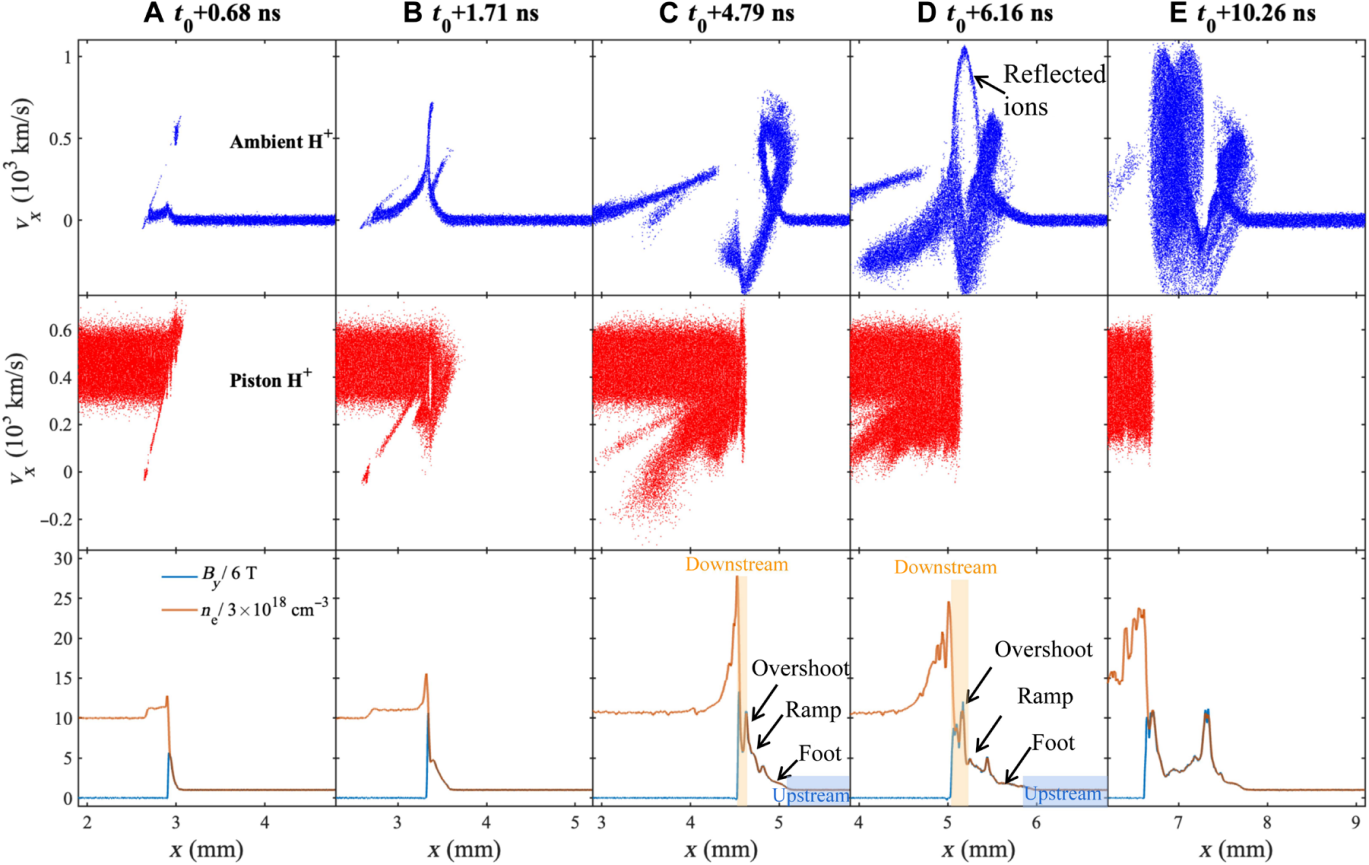
Formation of a shock structure and the associated ion dynamics in the 1D PIC simulation. The *v_px_*-*x* phase space scatterplots of the ambient (blue, first row) and piston (red, second row) H^+^ ions to present the ion dynamics associated with shock formation. (Third row) The magnetic field (blue) and the electron number density (red) profiles are displayed to show the formation of the piston-driven shock. The time steps of *t*_0_+0.68 ns (**A**), *t*_0_+1.71 ns (**B**), and *t*_0_+4.79 ns (**C**) correspond to the early time before shock formation, onset of shock formation (~ω*_ci–H_*^−1^ = 1.71 ns, which is the upstream H^+^ ion gyroperiod), and shock formation on ion scales that separated from the piston (*t* = *t*_0_+4.79 ns > 2ω*_ci–H_*^−1^), respectively. (**D** and **E**) Shock reformation after distinctly separating from the piston (see details in fig. S8). The proton-to-electron mass ratio is set as *m_p_*/*m_e_* = 100.

One-dimensional (1D) and 2D particle-in-cell (PIC) simulations are conducted to study the shock formation in piston-driven magnetized ambient plasma under conditions similar to our experimental parameters (see details in Materials and Methods), as illustrated in Fig. 2. At the beginning of the interaction, the piston acts like a snowplow with a speed of ~400 km/s and sweeps up the ambient ions and magnetic field (Fig. 2A), which produces density and magnetic field compression around the piston-ambient plasma interface. The particle trajectories indicate that the ions from the ambient and piston plasmas penetrate each other because the ions are effectively collisionless. Within *t*_0_+1.71 ns (ω_ci-*H*_^−1^ ~ 1.71 ns, the upstream H^+^ ion gyroperiod), the compressed steepened magnetic structure is strong enough to reflect the ambient H^+^ ions, at which time the shock begins to form (onset of shock; Fig. 2B) (*62*). After distinct separation from the piston, at approximately *t*_0_+4.79 ns, a shock on ion scales is formed with a speed of 415 km/s and *M_A_* ~ 8.3 (Fig. 2C). Consistent with our experimental results, the shock in the simulation reproduces the characteristic feature of a foot and a ramp, and the compression ratio is >3. In the following several gyroperiods, the shock reformation is observed in the shock foot region, and the C^5+^ ions form another shock behind the H^+^ ions shock (Fig. 2, D and E, and fig. S8 and S13).

**Fig. 3.**
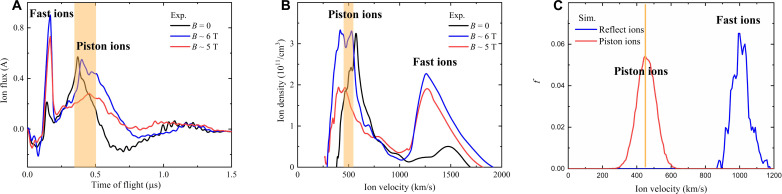
Ion velocity spectra in experiments and 1D PIC simulations. (**A**) TOF trace of ion flux in the experiments recorded by the Faraday cup along the symmetric axis of the piston plasma flow. After the precursor negative peak of the noise baseline (0 to 0.1 μs), the fast ions arrive at the Faraday cup first at ~0.16 μs, followed by the slow ions (piston) at ~0.4 μs. (**B**) Ion velocity spectra in the experiments that transform the TOF trace of ion flux [shown in (A)] to the collected ion density profile in a Faraday cup (see Materials and Methods and fig. S5). The slow ions with velocity *v* ~ 300 to 700 km/s come from the piston plasma. The fast ions with velocity *v* ~ 1100 to 1800 km/s, with approximately two to four times the shock speed, are the population from ambient ions accelerated by the shock. (**C**) Ion velocity spectrum collected in the foot region of the shock (*x* > 8 mm region at *t*_0_+11 ns; fig. S8) from the simulation with an external magnetic field of 6 T, which also exhibits two peaks. The velocity of the slow ions is ~400 km/s, whereas that of the fast ions is ~900 to 1200 km/s. The shock position is indicated by the orange shaded region.

Ion acceleration is observed in our experiments, accompanied by the formation of the magnetized collisionless shock. The TOF signal of ion flux (Fig. 3A), collected along the symmetric axis of the piston flow by the Faraday cup, presents two peaks in the ion velocity spectra (Fig. 3B). The first peak corresponds to the particles coming from the piston plasma, and the velocity is *v*_piston_ ~ 300 to 800 km/s, which is close to the shock speed (*v*_shock_ ~ 400 km/s). The second peak with the velocity *V*_fast_ions_ ~ 1100 to 1800 km/s, generated by the accelerated fast ions, which form the broad foot region (Fig. 1D), is found to have a quasi-monoenergetic spectrum and is approximately two to four times the shock speed, similar to the fast ion component observed in the Earth’s bow shock by satellites (*48*–*51*, *58*). When reducing the intensity of drive laser, the fast ion peak weakens until it is hidden by the piston ion signal. We have also changed the strength of the external magnetic field in the experiments and found that the fast ion peak becomes more pronounced with increasing external magnetic field (Fig. 3B). Even in the absence of external magnetic field, we still can observe the fast ion peak probably due to the self-generated magnetic field of ~1 T (see fig. S5 for further details) (*39*).

The PIC simulations of the experimental piston-ambient interaction, which also exhibit two peaks in the ion velocity spectra (Fig. 3C), confirm the ion acceleration capability of shock. The first peak of slow ions is provided by the piston plasma downstream of the shock. The second peak is the reflected fast ions in upstream with approximately two to three times the shock speed. H^+^ ions picked up from the ambient plasma dominate the fast ions and are accelerated during reflection by the shock (see section S4). Shock formation and ion acceleration are not observed in simulations with approximately zero external magnetic field. Notably, the detailed characteristics of the ion velocity spectra in our simulation cannot be straightforwardly compared with experiments for the following reasons. First, the experiments results are temporally and spatially integrated with ions escaping from the 2D hemispherical shock with an inhomogeneous background profile, although the simulation is just a 1D or 2D homogeneous background with reduced proton-to-electron mass ratio to lessen computational burden. Second, the magnetized ambient plasma has a finite size of ≤10 mm in experiments (Fig. 1 and fig. S1). Thus, the reflected ambient ions can escape into vacuum and move ballistically into detector, before gyrating back into downstream, when the shock reaches the boundary of the magnetized ambient plasma (see Materials and Methods and fig. S6 for further details), although the simulation is in situ measurement.

**Fig. 4.**
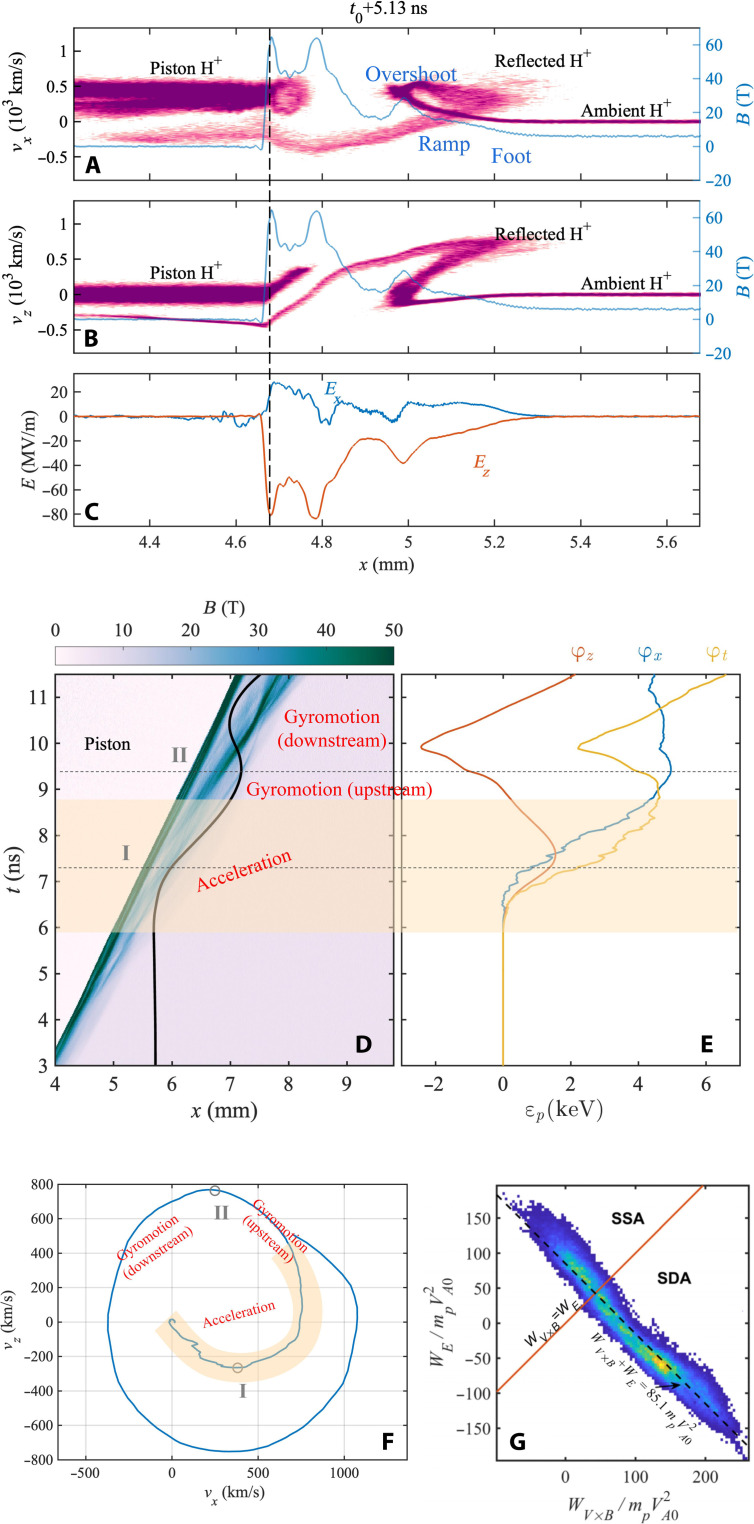
Ion acceleration in 1D PIC simulations. (**A** and **B**) *v_px_*-*x* (A) and *v_pz_*-*x* (B) phase space scatterplots of the H^+^ ions at *t*_0_+5.13 ns (normalized, including ambient and piston plasma), along with the profile of the magnetic field (blue line). (**C**) *E_x_* (blue) and *E_z_* (red) electric fields at *t*_0_+5.05 ns. (**D**) Trajectory (black) of a typical reflected H^+^ ion originating from ambient plasma overlaid on the profile of the magnetic field strength (color bar). (**E**) Time history of the potential gain of the reflected H^+^ ion φ*_x_* (olive), φ*_z_* (pink), and φ*_t_* (black) ($\varphi_{i}=\int_{t}E_{i}v_{i}\mathrm{dt},i=x,z$, and the total potential gain $\varphi_{t}=\varphi_{x}+\varphi_{z}$). (**F**) H^+^ ion trajectory in the *v_z_*-*v_x_* space. The external magnetic field *B_y_* is 6 T. The interface between the shock and piston is labeled approximately with the dashed line in (A) to (C). In (D) to (F), the reflection and acceleration stage is indicated by the orange shaded region, whereas the moments of ion reflection and that ion gyrates back into downstream are labeled with lines/circles I and II, respectively. (**G**) *W_E_*-*W_V×B_* diagram of the reflected ions (ω*_ci–H_t* = 3~6) to show the dominated mechanism of SDA. *W_E_* and *W_V×B_* are the work done by the electric force and Lorentz force in the +*x* direction during reflection in the shock transition layer before entering the upstream region.

Figure 4 illustrates the simulated ion dynamics, demonstrating that there exist two components of accelerating electric fields *E_x_* and *E_z_* associated with the shock (Fig. 4, A to C). The electric field *E_x_* is an electrostatic field caused by motional electric field and charge separation, whereas the electric field *E_z_* is only a motional electric field (*62*, *63*) (~*v*_shock_*B*_d_, where *B*_d_ is the magnetic field downstream). By following the trajectory of a randomly chosen typical single reflected H^+^ ion described in Fig. 4 (D to F), we can identify that the particle energization around the shock, which is dominated by the motional electric field (fig. S9), can be approximately separated into two stages. In the first stage of “reflection and acceleration” (the orange shaded region in Fig. 4D), the H^+^ ion slides into the shock foot (~6.0 ns) and gets accelerated by the *E_x_* and *E_z_* field. At ~7.2 ns, the H^+^ ion is reflected toward upstream, followed by further acceleration until escape from the shock transition layer into the upstream region. Then, the reflected H^+^ ion starts the second stage of “gyromotion” at ~8.7 ns in the upstream region with little energization. Subsequent to energization, part of the reflected H^+^ ions gyrate into the downstream region and dissipate energy in it, whereas the remaining H^+^ ions are still in the upstream region, which can escape into vacuum when the shock moves to the boundary of the magnetized ambient plasma of finite size (fig. S6) and produces the quasi-monoenergetic fast ion peak collected by the Faraday cup in our experiments. Assuming that the acceleration timescale in the motional electric field is approximately one gyroperiod *m*/(*qB*_ave_) (*B*_ave_ is the average magnetic field that the reflected ions are experienced around the shock), the velocity gain of the reflected ions in the *z* direction can be estimated as Δvz∼vshockB/dBave∼(1−3)vshock. Therefore, the reflected ions have a speed of approximately $v∼\sqrt{\Delta v_{x}^{2}+\Delta v_{y}^{2}}∼\left(1.4-3.2\right)v_{\text{shock}}$, consistent with our experiments.

To elucidate the dominated acceleration mechanism, we analyzed the works (*20*, *46*) done by the electric force and the Lorentz force in the +*x* direction during ion reflection in the shock transition layer before entering the upstream region. It shows that 73% of energetic ions undergo SDA (Fig. 4G), whereas SSA only contributes a small share. Our results confirm that SDA dominates the ion energization in the Earth’s bow shock (*41*–*45*, *47*–*51*), not SSA claimed previously (*40*). Different from SSA that the *E_z_* electric field along the shock front dominates acceleration (*20*, *40*, *46*), ions are energized by both *E_x_* and *E_z_* electric field in SDA (*44*). Therefore, we can measure intense fast ion flux in the +*x* direction, facing the shock front rather than along the shock front (along magnetic field lines) in previous experiments (*40*).

Our 1D and 2D simulations indicate that the reflection efficiency of the ambient ions is about 20 to 26%, and most of the accelerated ions are H^+^ (C^5+^ ions ratio is less than 1%). More effective reflection of SDA with middle Mach number shock in our experiments can interpret (*41*, *42*, *44*) the energetic ion flux of three to four orders of magnitude higher than the experiment of low–Mach number shock (*40*). The high-energy tail of 50 to 100 keV observed in the previous experiment (*40*) can be attributed to the fast ion component with two to three times the shock speed predicted by SDA, whereas the low-energy portion of 20 to 50 keV in that experiment probably comes from the downstream, where the energetic ion has dissipated part of its energy.

We found that a small fraction (<0.1%) of the earlier reflected ions can undergo multiple reflections and acceleration between upstream and shock front, producing higher energy ions with a continuous spectrum that ends up in the downstream region (fig. S11) and potentially start the Fermi energization cycle. The higher-energy ions are three orders of magnitude weaker than the quasi-monoenergetic fast ion peak in our experiments; thus, it will be hidden under our experimental noise baseline.

## DISCUSSION

In conclusion, our results provide the direct laboratory experimental evidence of ion energization from single reflection off a supercritical quasi-perpendicular collisionless shock, which are consistent with the satellite observations of the quasi-monoenergetic fast ion component in the Earth’s bow shock (*48*–*51*, *58*). We identify that SDA dominates the ion energization in the Earth’s bow shock, not SSA claimed previously. It has more effective reflection, and both electric field components along shock’s normal and tangential directions attribute to the ion acceleration. Repeated reflections from collisionless shock, accompanied by successive small energy increments, have the potential to push charged-particle energies up to very high values for initiating the Fermi acceleration cycle and producing the high-energy charged particles in the Universe. The electron acceleration should also be experienced in this process, which is the task we will explore in the future experiments, whereas the parallel shock is still a challenge for laser-driven collisionless shock (*18*, *64*, *65*). This paves the way for controlled laboratory experiments that can greatly complement remote sensing and spacecraft observations and help validate particle acceleration models. Our results are of benefit to the laser-driven ion accelerator (*66*), which indicates that adding a magnetic field can potentially increase the energetic ion dose markedly. Our observation can also provide a useful guidance for laser fusion, at which collisionless shock via the self-generated magnetic field (*67*–*70*) will produce energetic ions and perturb the capsule compression seriously (*71*).

## MATERIALS AND METHODS

### Experimental setup

Experiments were conducted at the Shenguang-**II** laser facility at Shanghai Institute of Optical and Fine Mechanics of Chinese Academy of Sciences. A pair of plastic CH_2_ planar foils (200-μm-thick, 2-mm by 2-mm square foil) was used as the ambient and piston targets, which were separated by 6 mm in the *z* direction (horizontal) and 3 mm in the *x* direction (vertical). An external imposed magnetic field *B_y_* is generated by magnetic field coils with a Φ10-mm inner diameter (*52*), which are placed ~1 mm away from the planar foils. As shown in fig. S1, which is a top view of the experimental setup, both targets were embedded in a quasi-uniform magnetic field of ~5 to 6 T, which lasts ~200 ns (flat-top width of pulsed magnetic field with >95% of peak strength). The left “ambient” target, which created an ambient plasma, was heated by a low-energy precursor laser beam with a 100-J energy, 1-ns square pulse duration, and 351-nm wavelength. The precursor laser beam passed through a beam-smoothing phase plate, tiled, and defocused over the surface of the ambient target to produce a flat-topped intensity distribution over the central 0.8-mm by 1.0-mm square region, resulting in an average intensity of ~1 × 10^13^ W/cm^2^. Twelve nanoseconds later, at time *t*_0_, the ambient plasma was magnetized by an external magnetic field (*39*, *54*–*56*), and an intense drive laser beam (~8 × 10^13^ W/cm^2^; 260 J, 1 ns, 351 nm, and 0.5-mm by 0.5-mm square smoothed focus spot) irradiated the top “piston” target to generate a supersonic plasma flow, which expanded into the magnetized ambient plasma and drove a magnetized collisionless shock.

### Optical diagnostics

The shock structure and the electron density profiles of the ambient plasma were characterized using a probe laser beam of 527-nm wavelength and 80-ps pulse duration that passed through the plasma along the *y* direction, as shown in fig. S2, producing simultaneous images of the optical interferometry and the dark-field schlieren method. Optical interferometry measures the line-integrated electron density profile. Whereas the dark-field schlieren method measures the first spatial derivative of the line integrated electron density profile, the bright refractive fringes indicate the discontinuity surfaces around the shock. As shown in Fig. 1B and fig. S3, the bright refractive fringes of dark-field schlieren images overlap perfectly with the splitting or severely twisted fringes of optical interferometric images, which clearly indicate the location and speed of the shock. The measured shock speeds are ~400 km/s for magnetized shock at *B_y_* ~ 5 T, which is less than that without an external magnetic field of 500 km/s but still within the measurement error, as shown in fig. S4.

### TOF measurements of the ion velocity spectrum

The ion velocity spectrum in the experiment was measured by the TOF method using a Faraday cup (Kimball physics model FC71A), which was placed below the piston target at a distance of 21 cm (along the *x* direction). A grid voltage of −60 V is supplied to repel electron injection. The collector and injection hole of the Faraday cup cast a view field of Φ5-mm diameter over the plasma region, which covers most of the shock region. Ions with speeds higher than 2000 km/s will be hidden under the precursor negative peak (0 to 0.1 μs), which may be caused by ultraviolet light and soft x-rays. Although electromagnetic shielding was used on the Faraday cup, there were still observable low-frequency noises in some shots, as shown in fig. S5, which may disturb the slow ion signal coming from piston plasma flow. Ion velocity and kinetic energy spectra can be transformed from the TOF signal of ion flux (section S3).

### 1D PIC simulations

A fully electromagnetic, full relativistic PIC code (*72*) is used to simulate the interaction of piston plasma flow with magnetized ambient plasma. The simulations have one spatial dimension, but the particles have 3D velocity components (1D3V). Two species consisting of 1:1 mixed C^5+^ and H^+^ ions are set for both the piston and ambient plasma. We initialize the simulation domain with a box size of $L_{x}=80c/\omega_{\mathrm{pi}}=10.56\;\text{mm}$ (where *c* and $\omega_{\mathrm{pi}}=\sqrt{\frac{n_{\mathrm{ae}}e^{2}}{m_{p}\varepsilon_{0}}}$ are the light speed and H^+^ ion plasma frequency, respectively), and the size of one cell is $\Delta x=0.02c/\omega_{\mathrm{pi}}=2.64\;\mu m$, which is smaller than the Debye length $\lambda_{D}=0.0866c/\omega_{\mathrm{pi}}=11.43\;\mu m$ of the ambient plasma. One hundred macroparticles are distributed in one cell for electrons in the ambient plasma, and second-order shape function is used to describe the macroparticle. The light speed *c* is set to 73.6*v_A_* (where the Alfvénic velocity *v_A_* is calculated based on $v_{A}=B_{0}/\sqrt{\mu_{0}\left(m_{p}n_{\mathrm{ap}}+m_{c}n_{\mathrm{ac}}\right)}=51.29\;\text{km}/s$), and the proton-to-electron mass ratio is reduced to *m_p_*/*m_e_* = 100 to lessen computational burden. It shows that the simulation results of *m_p_*/*m_e_* = 100 are close to that of the real mass ratio of *m_p_*/*m_e_* = 1836 (*38*, *73*, *74*). The ambient plasma, embedded in a magnetic field of *B_y_* = 6 T, has electron number density $n_{\mathrm{ae}}=3\times 10^{18}/{\text{cm}}^{3}$ and temperature $T_{C}=T_{H}=T_{e}=30\;\text{eV}$ and is filled initially in the region of $20.23c/\omega_{\mathrm{pi}}<x<80c/\omega_{\mathrm{pi}}$. The piston plasma, located initially in the region of $0<x<20c/\omega_{\mathrm{pi}}$ with a bulk velocity of 453 km/s along the +*x* direction, has a uniform electron number density $n_{\mathrm{pe}}=10n_{\mathrm{ae}}$ and temperature $T_{C}=T_{H}=T_{e}=800\;\text{eV}$. The plasma density decreases linearly from *n_pe_* to *n_ae_* in the transition region of $20c/\omega_{\mathrm{pi}}<x<20.23c/\omega_{\mathrm{pi}}$ .The time step is $\Delta t=2\times 10^{-5}\omega_{\mathrm{ci}-H}^{-1}$ (where $\omega_{\mathrm{ci}-H}$ is the ambient proton gyrofrequency). As shown in Fig. 2, a quasi-perpendicular shock is formed in the magnetized ambient plasma around $t=2.0\omega_{\mathrm{ci}-H}^{-1}$ (~3.42 ns) driven by piston plasma flow and propagates with a velocity of 350 to 480 km/s, which leads to an Alfvénic Mach number *M_A_* = 6.8 to 9.4.

### 2D PIC simulation

A quasi-2D piston flow–driven shock is simulated with the fully kinetic relativistic parallel PIC code OSIRIS 4 (*75*). The sketch of simulation setup is shown in fig. S13. The simulation used a thin rectangular domain of *L_x_* = 1020 *c*/ω*_pe_* and *L_y_* = 1 *c*/ω*_pe_* in the *x*-*y* plane (ω*_pe_* is the electron plasma frequency), with 40,800 and 40 grids in the *x* and *y* directions, respectively. Quartic particle shape function is applied. Also, the time step is d*t* = 0.0175/ω*_pe_*. An open boundary condition is used in the *x* direction for both particles and fields, whereas a periodic boundary condition is applied in the *y* direction. Reduced ion masses are used in the simulation where *m_H_*/*m_e_* = 100 and *m_C_*/*m_e_* = 1200. The piston plasma flow with a 200-eV temperature is initialized in the region *x* > 824 *c*/ω*_pe_*, whereas the ambient plasma with a 40-eV temperature is at rest in the region of *x* < 824 *c*/ω*_pe_*. Both piston and ambient plasmas are composed of C^5+^ ion, H^+^ ion, and electrons, with a number density ratio of 1:2:7. The electron densities of ambient and piston plasma are *n_ae_* = 3 × 10^18^ cm^−3^ and *n_pe_* = 10*n_ae_*, respectively. An in-plane *B*-field of *B_y_* = 6 T is applied in the ambient plasma along the *y* direction, perpendicular to the shock normal. This setup gives Alfvénic velocity $v_{A}=B_{0}/\sqrt{\mu_{0}\left(m_{p}n_{\mathrm{ap}}+m_{c}n_{\mathrm{ac}}\right)}=228\;\text{km}/s$. The piston flow drifts to the left (negative *x* direction) with an Alfvénic Mach number of 8.5, driving a shock that propagates to the left. The Coulomb collision module is disabled in the simulation. The simulation results are shown in fig. S14. At *t* = 1.02ω*_ci–H_*^−1^ (the H^+^ ion gyroperiod), the upstream ions begin to reflect. Then, at *t* = 2.38ω*_ci–H_*^−1^ (the C^5+^ ion gyroperiod), both the C^5+^ and H^+^ ions form shock structures, and the shock of C^5+^ moves slower behind of the H^+^ shock. Last, at *t* = 4.76ω*_ci–H_*^−1^, the shocks separate from the piston substantially. The 2D simulation results agree well with the 1D simulation results.

### Discrepancy between remote ion detection used in experiment and in situ ion measurement used in spacecraft and simulation

The simulations indicate that the reflected ions gyrate only for finite duration in the upstream region and will transmit into downstream eventually and dissipate their energy, which probably means that the reflected ions can only be in situ measured as the satellites in space, and it is unlikely to be observed by the remote detector used in our experiments. This confusion comes from the finite size of the magnetized ambient plasma in experiments (fig. S6). The current-carrying coils in our experiments can only impose a magnetic field of 10 mm in size in the shock movement direction, and the laser ablation produced ambient plasma expands the similar size in the ~20-ns experimental period. Beyond the Φ10-mm current-carrying coils is the near-vacuum region without a magnetic field. Thus, when the shock moves to the boundary of the magnetic field, the reflected ions will escape into vacuum before gyrating back into downstream and move ballistically into the remote ion detector of the Faraday cup (even in the ambient plasma, the ion-ion collisional mean free path of fast ion with a velocity of 1200 km/s is more than 320 mm, which is much larger than the plasma size of ~10 mm).

The remote detection ensures that the recorded reflected ions come from the quasi-perpendicular shock at the equator of the hemispherical shock. Because the Faraday cup opens just ±0.7° view angle toward the shock along the −*x* direction, only the reflected ion with velocity dominantly in the *x* direction can enter the detector. The reflected ions generated by shock off the equatorial plane have a non-negligible *y*-direction velocity component and cannot enter the Faraday cup. Therefore, the recorded reflected ions come from the quasi-perpendicular shock (90° ± 0.7°) at the equator of the hemispherical shock. It also implies that the detected ions originate from a local position on the equator of the shock and that the *y* and *z* components of the velocity are negligible. Thus, it can be assumed that they came from reflection off the quasi-1D quasi-perpendicular part of the shock.

## Supplementary Materials

### This PDF file includes:

Supplementary Text Figs. S1 to S14 References
