# Supplementary material for: Laboratory observation of ion drift acceleration via reflection off laser-produced magnetized collisionless shocks
Source: Sci Adv. 2025 Feb 12;11(7):eadn3320. doi: 10.1126/sciadv.adn3320 (PMC11818023; doi:10.1126/sciadv.adn3320)
Supplement: Supplementary file 1 — Supplementary Text Figs. S1 to S14 References [file sciadv.adn3320_sm.pdf]

Science Advances
AAAS

Supplementary Materials for

Laboratory observation of ion drift acceleration via reflection off laser-produced magnetized collisionless shocks

Hui-bo Tang *et al.*

Corresponding author: Guang-yue Hu, gyhu@ustc.edu.cn; Quan-ming Lu, qmlu@ustc.edu.cn

Sci. Adv. **11**, eadn3320 (2025)
DOI: 10.1126/sciadv.adn3320

This PDF file includes:

Supplementary Text
Figs. S1 to S14
References

S1. Supplementary for experimental setup

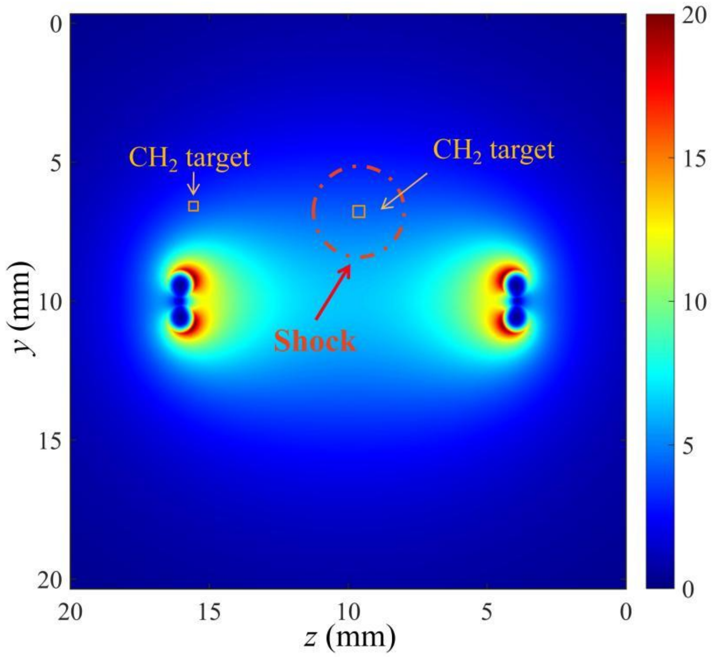

Fig. S1: **Top view of the experimental setup**. CH_2 targets and shock structure were embedded in a quasi-uniform magnetic field of about 6T. The contours of magnetic field were provided by simulation *(52)*.

S2. Supplementary for optical diagnostics

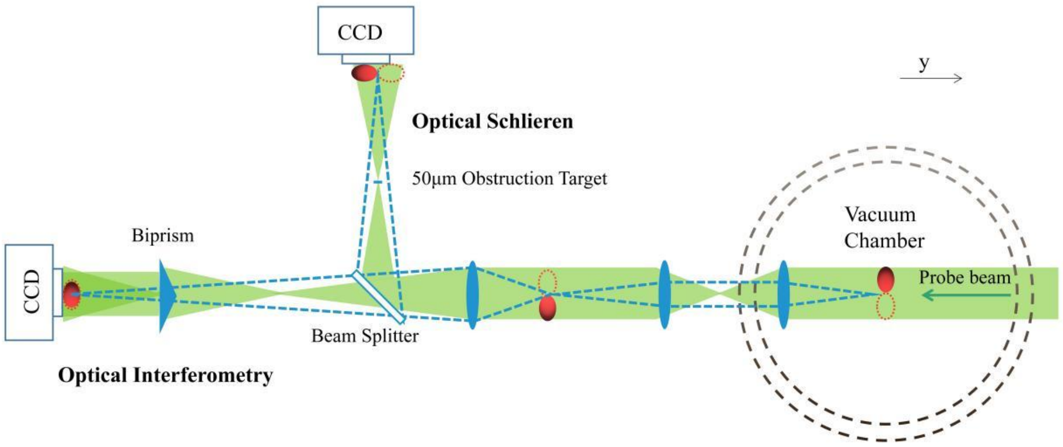

Fig. S2. Sketch of optical diagnostics to measure the shock structure and the ambient plasma density. After passing through the plasma along y direction, the 80ps/ 527nm probe laser beam was split into two paths by a beam splitter. One path produces the image of optical interferometry via a biprism. The other path generates the dark-field schlieren image which using an obstruction target of Φ50μm diameter to block the light directly from the source (dark-field) while pass through the refracted light by the discontinuity surfaces around the shock.

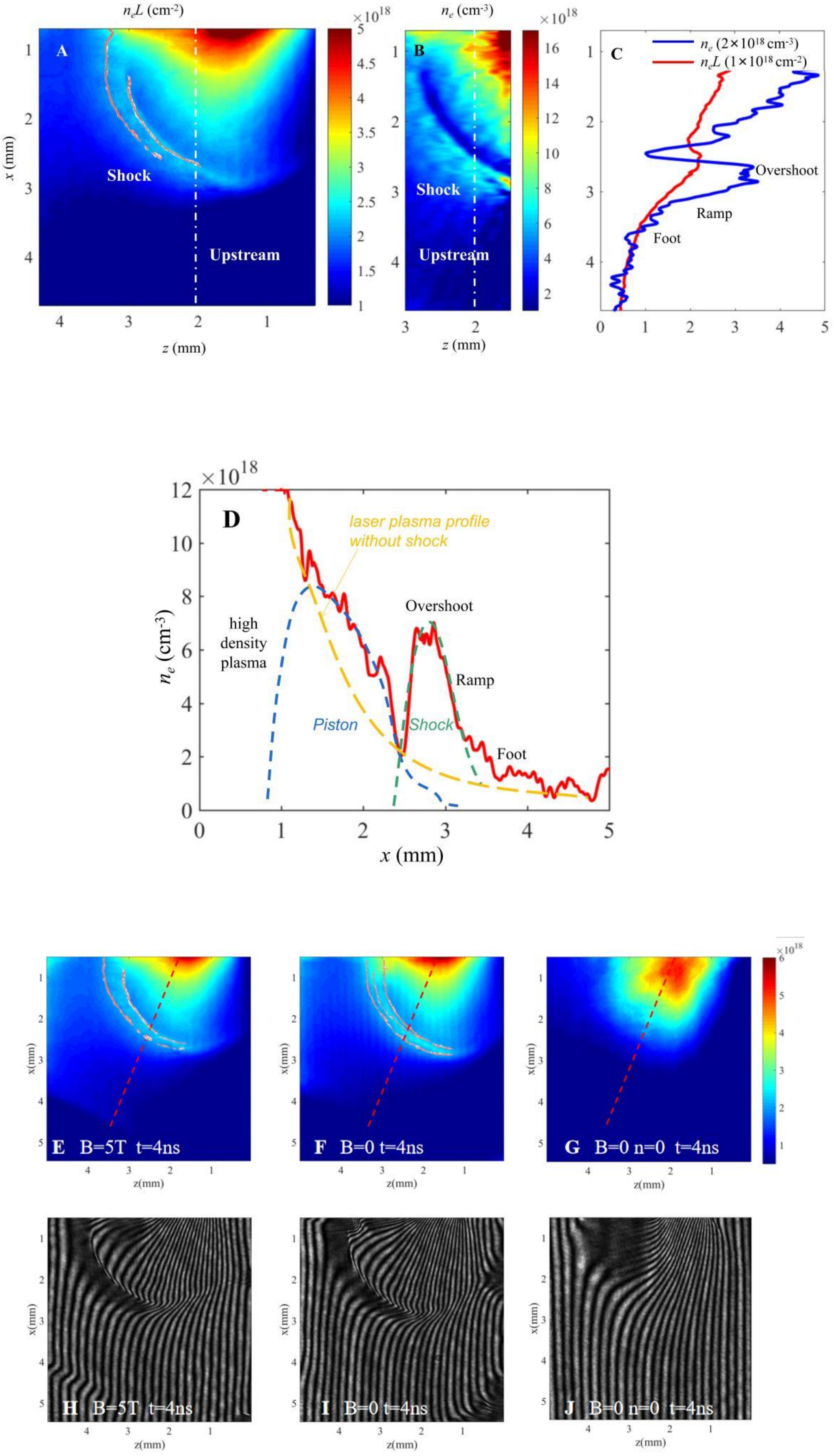

n_eL (cm^{-2})
A
Shock
Upstream
x (mm)
z (mm)
×10^{18}
n_e (cm^{-3})
B
Shock
Upstream
z (mm)
×10^{18}
C
n_e (2×10^{18} cm^{-3})
n_eL (1×10^{18} cm^{-2})
Overshoot
Ramp
Foot
×10^{18}
D
laser plasma profile
without shock
high
density
plasma
Overshoot
Ramp
Piston
Shock
Foot
n_e (cm^{-3})
x (mm)
E B=5T t=4ns
F B=0 t=4ns
G B=0 n=0 t=4ns
×10^{18}
x(mm)
z(mm)
H B=5T t=4ns
I B=0 t=4ns
J B=0 n=0 t=4ns

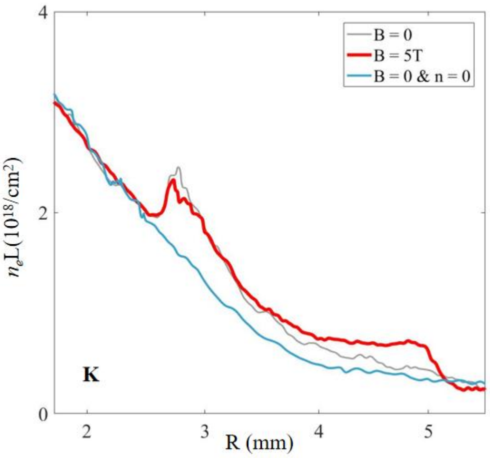

Fig. S3. Optical images of collisionless shock at 4ns with external magnetic field of 5T. (**A**) 2-D line-integrated electron density distribution given by the optical interferometry. The bright refractive fringes in the images of dark-field schlieren method (red contour lines) are overlapped perfectly with the position of shock in the 2-D electron density profile, which clearly indicates the location and the speed of shock. (**B**) Electron density profile obtained by Abel inversion if assuming a cylindrical symmetric density distribution around the z=1.5mm line. It doesn't mean to quantify since the shock is asymmetric actually, but to highlight the shock structure. (**C**) The shock profile along the z=2mm line taken from (**A**) and (**B**) respectively. In each images, the piston target is located at x=0mm, while the ambient target is located outside the left borders. *L* is the plasma size in y direction. (**D**) Sketch of the collisionless shock structure. **(E-G)** the line-integrated electron density profiles (color bar) and the schlieren contours (red, produced by density gradient around the shock) for the cases of B=5T , B=0, and B=0 without ambient plasma (B=0 & n=0, only piston). **(H-J)** the fringes of the optical interferometry for the cases of B=5T , B=0 and B=0 without ambient plasma. (**K**) the 1-D line-integrated density profiles taken along the red line in (E-G) for the cases of B=5T, B=0, and B=0 without ambient plasma (B=0 & n=0, only piston).

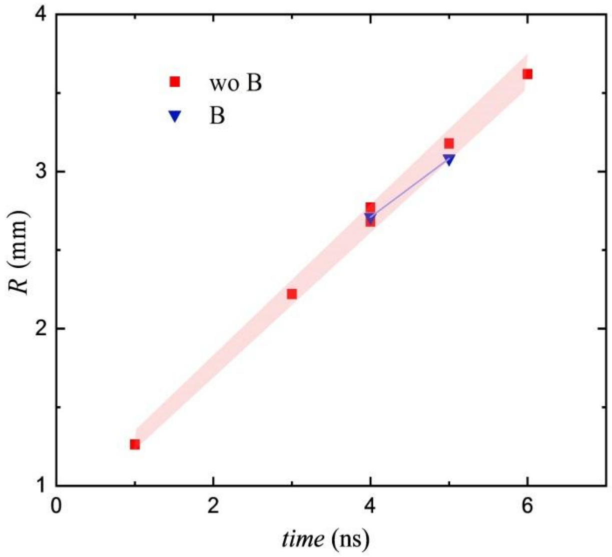

Fig. S4. The shock position and fitted speed measured by optical diagnostics. *R* is the distance between the shock's discontinuous surface and the piston target. The shock speed of 400km/s at 5T external magnetic field (blue) is less than that without external magnetic field of 500km/s (red) but still within measurement error. Discontinuous surfaces of shocks are not formed before 1ns, during which the piston plasma experiences the initial free expansion phase with very high speed.

S3. Supplementary for measurement of ion velocity spectrum in experiment

(a) Measurement of ion velocity spectrum

The Faraday Cup along the symmetric axis of the piston plasma flow measures the time-of-flight trace of ion flux in the experiments. Then the time-of-flight trace of ion flux was transformed into ion density profile collected by Faraday cup, which is the ion velocity spectrum. It is assumed that protons dominate the ions that enter the Faraday Cup in the calculation of the ions velocity spectrum, although there are a small amount of Carbon ions.

The ions velocity spectrum (ion density profile) can be obtained from Eq. (S1) *(77)*, as shown in Fig. S5b

$$n_i = \frac{I}{qSv_i} \tag{S1}$$

where *I* is the electric current of collected ions recorded by oscilloscope (Fig. S5a), n_i is the ions number density collected by Faraday Cup, *q* is the ions charge state, S is the collector area of Faraday Cup, and v_i is the ion velocity, and

$$vi = \frac{L^*}{t_{TOF}} = \frac{21cm}{t_{TOF}} \tag{S2}$$

where L^* is the distance of Faraday Cup relative to target, and t_{TOF} is the time-of-flight of ion.

The ions kinetic energy spectrum dN/dE_k can also be obtained from the electric current signal of collected ions. The ions yield is

$$N = \frac{Q}{q} = \frac{\int I(t_{TOF})dt_{TOF}}{q} \tag{S3}$$

where Q is the total charge of collected ions in Faraday cup. And the ion kinetic energy is $E_k = \frac{1}{2}m_i v_i^2 = \frac{1}{2}m_i(\frac{L^*}{t_{TOF}})^2$, so dN/dE_k can be obtained by taking the derivative of the ion yield *N,* as shown in Fig. S5C.

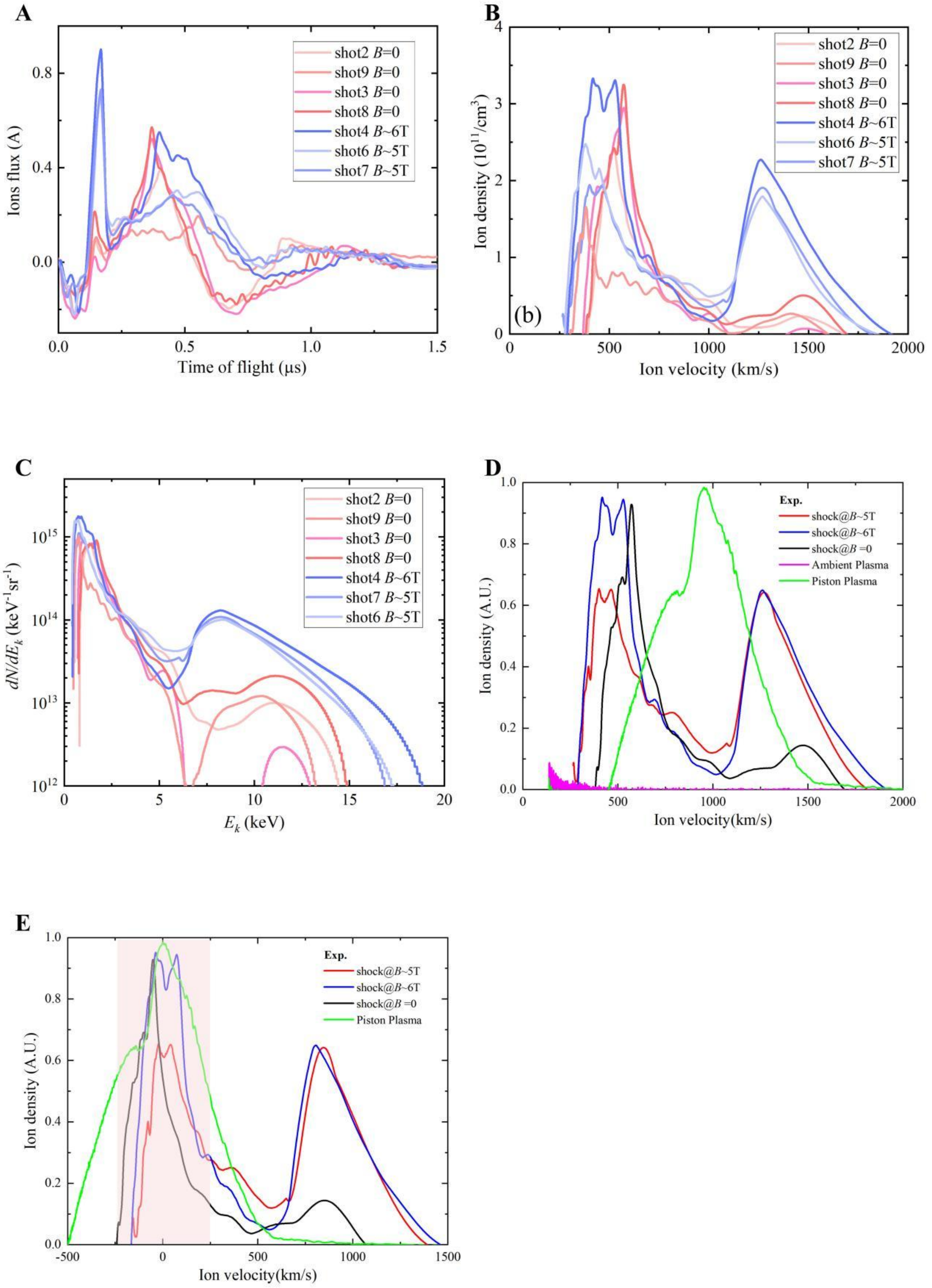

Fig. S5. Additional signals of ion velocity spectrum measured by Faraday cup in experiments. (**A**) Time-of-flight trace of ion flux. Fast ions arrive the Faraday Cup at 0.16μs, followed by slow ions around 0.4μs. (**B**) The ions velocity spectrum. The slow ions around 300-800km/s are close to the shock's speed of 400km/s. The fast ions

around 1100-1800km/s are ambient H^+ ions accelerated by the shock, which is 2-4 times of the speed of shock. (**C**) The ions kinetic energy spectrum. The fast ions are peaked in the range of 6-18keV, which is one order of magnitude higher than that without external magnetic field, and 3-4 orders of magnitude higher than the high-energy continuous spectrum previously observed in the region of 20-80keV *(56)*. (**D**) The ion velocity spectrum in the laboratory frame. (**F**) The ion velocity spectrum in the piston-rest frame.

(b) Sketch of reflected ion escaping into vacuum when the shock moves to the boundary of magnetized ambient plasma with finite size of ~10mm

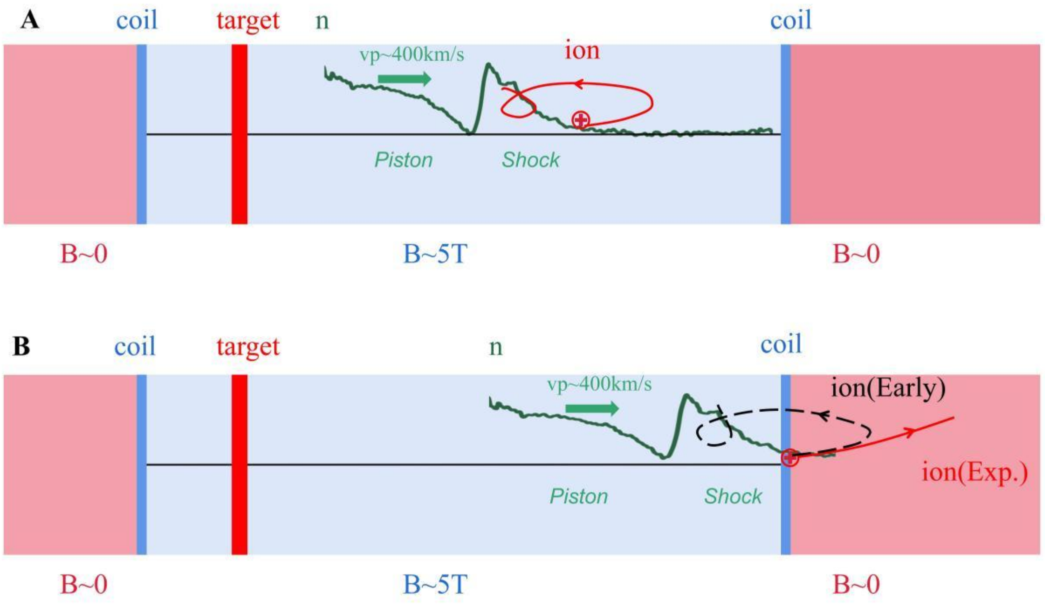

Fig. S6. Sketch of the reflected ions trajectory in the magnetized ambient plasma with finite size. (**A**) Early reflected ions can be eventually transmitted into downstream region and dissipate its energy (such as at ~3ns). (**B**) Late reflected ions will escape into vacuum when the shock moves to boundary of the Φ10 mm current-carrying coils (for example at ~12ns), and enter the remote ion detector of Faraday cup. Notice in this version the piston and shock move toward right direction.

S4. Supplementary for PIC simulation

(a) Dynamics of typical single-reflected ion.

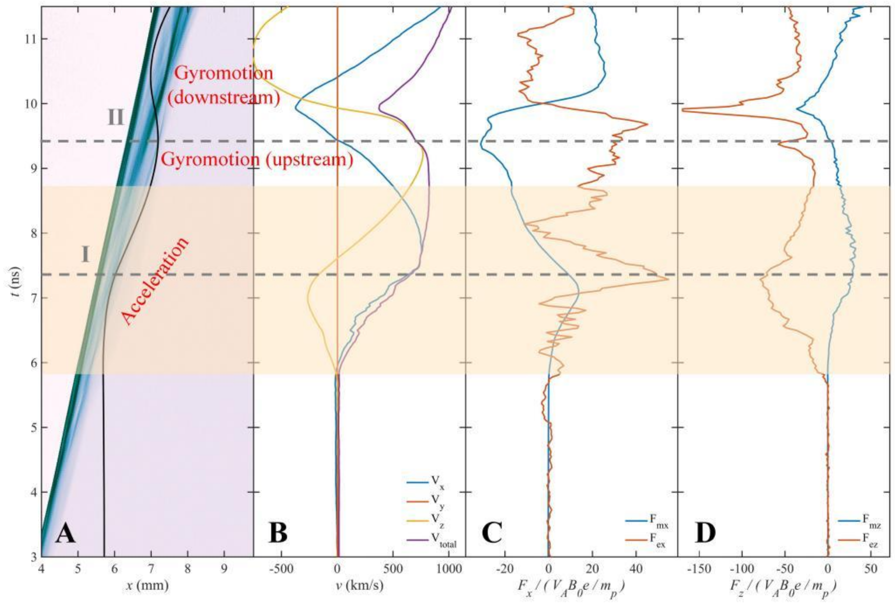

Fig. S7. Ion dynamics of typical single-reflected H^+ ion in 1-D PIC simulation with m_p/m_e=100. (**A**) The ion trajectory and the shock evolution of magnetic field B_y. (**B**) The ion velocity gain in x-, y, z- direction. (**C-D**) The electric and Lorentz forces in *x*-, and *z*- direction respectively. After the ion drifting into shock transition layer, the acceleration process starts, and continues after reflection until running back to the upstream. After a cycle of gyration in the upstream region, ion return to the downstream region, trapped in it to dissipate energy. Orange shaded region indicates the acceleration stage.

(b) The ambient ions experience the magnetically reflection and form a shock

As shown in the Fig. S8, ambient and piston ions penetrate to each other at first (t=0.68ns, Fig. S8**A**). The piston, with the speed of 400km/s, sweeps up the ambient ions and magnetic field, which produces density and magnetic field compression around the piston-ambient plasma interface. At t=1.71*ns* (Fig. S8**B**), the compressed steepened magnetic structure is strong enough to result in the reflection of ambient H^+ ions. As the ambient H^+ ions being reflected, a shock front is formed and begins to separate from the piston at t=3.42ns (~$2\omega_{ci\text{-}H}^{-1}$, Fig. S8**C**). Distinct separation of the shock from the piston can be observed at t=4.45ns with characteristic feature of a "foot", a "ramp", and an "overshoot" (Fig. S8**D**). The reflected ambient H^+ ions are obviously accelerated in x and z directions. While the initial accelerated piston ions were reflected back into the piston region and cannot be accelerated by the shock. After 6ns (Fig. S8e-g, and Fig. 4**D**), a new ramp starts growing in the foot region which exhibits as reformation of the shock profile. As expected *(39)*, C^{5+} ion shock is formed behind the H^+ ions shock with the characteristic time scale of C^{5+} ion gyroperiod $\omega_{ci\text{-}C}^{-1}$ (Fig. S8**D**).

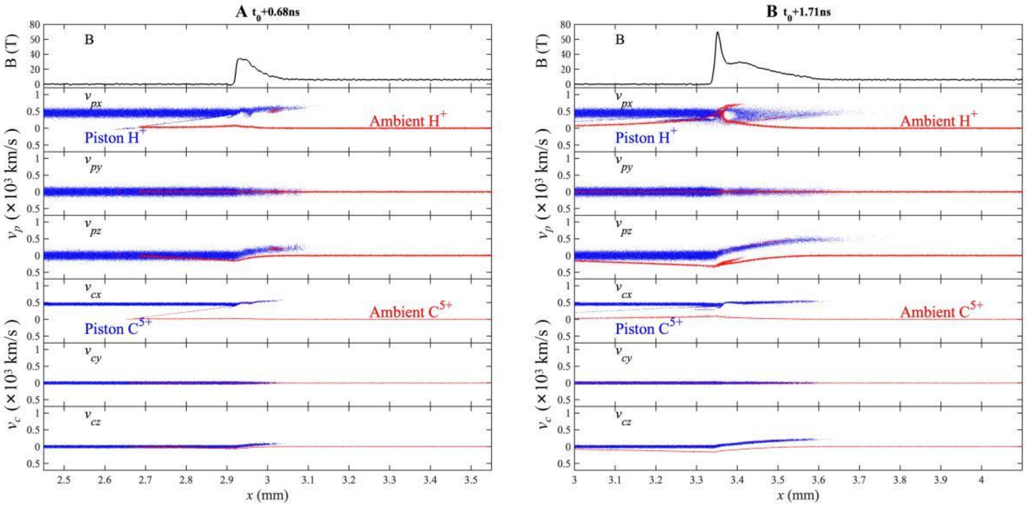

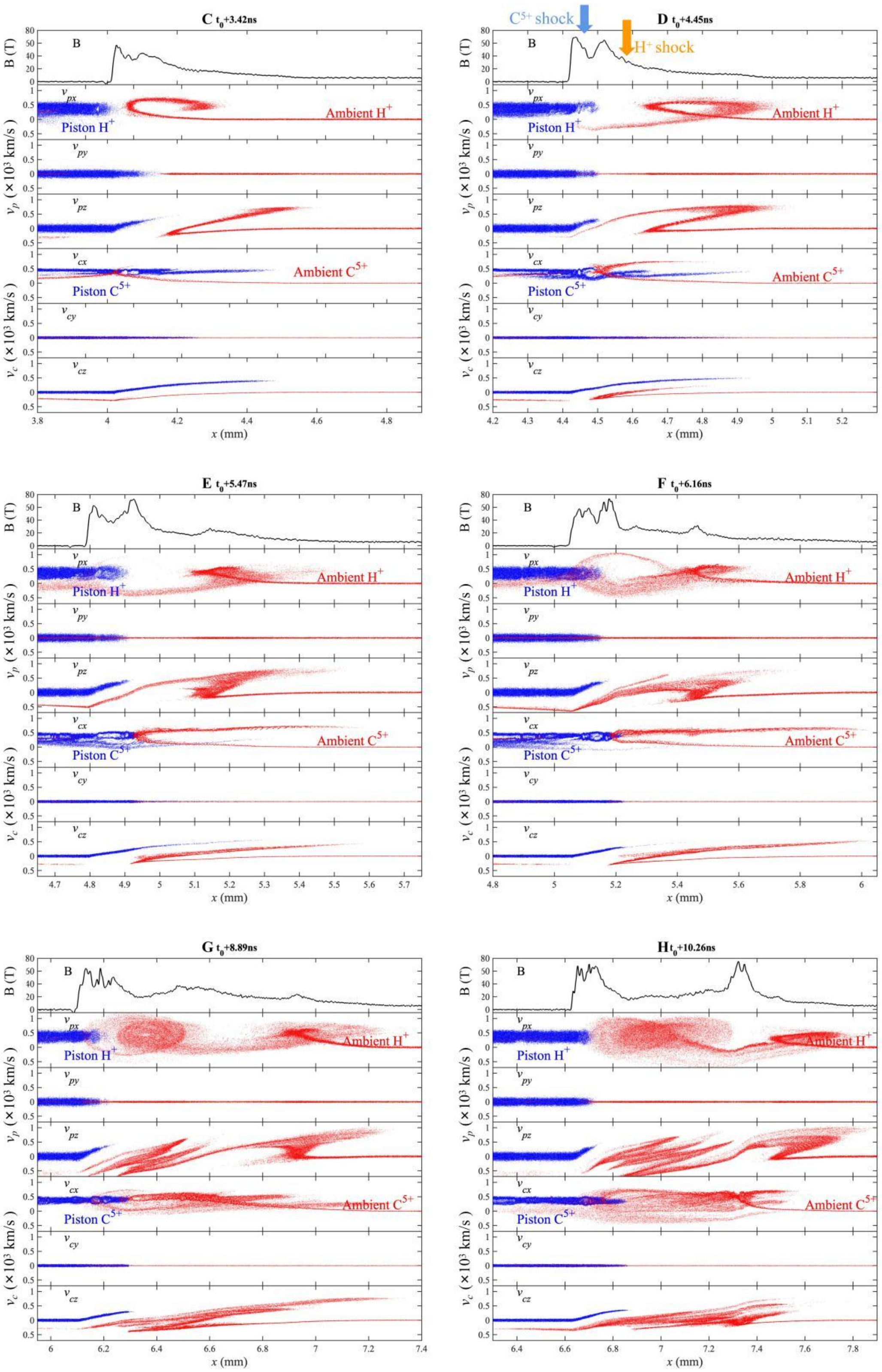

Fig. S8. Ion dynamics of H^+ and C^{5+} ions during shock formation in 1-D PIC simulation from 0.68ns to 10.26ns (A - G). The magnetic fields profile B_y, ambient and piston ion phase space ($v_x/v_y/v_z$, x) are displayed from top to bottom rows.

(c) The reflection and acceleration of ions are dominated by motional electric field and shock drift acceleration (SDA)

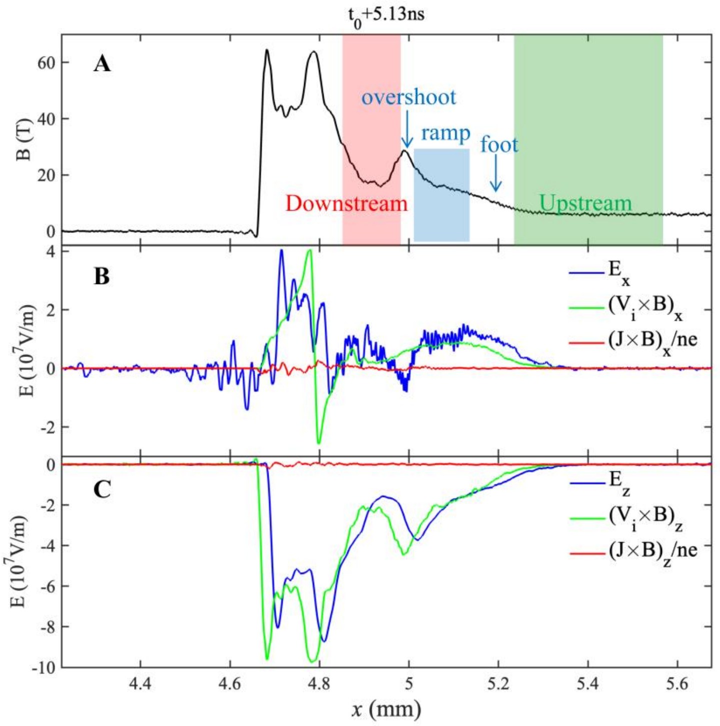

Fig. S9. Profiles of magnetic field B_y (A), electric field components E_x (B), and E_z (C) associated with the shock at t_0+5.05ns in simulations in the frame of laboratory. Electric field terms (blue) in the generalized Ohm's law are displayed in (**B-C**) including motional electric field/ion Lorentz term $V_i \times B$ (green), Hall term $J \times B$ (red), and electron pressure term $\nabla \cdot P_e$ (equal to E-$V_i \times B$-$J \times B$, not shown). Motional electric field are dominant for E_x and E_z *(78)* in the shock transition layer, where ambient ions are reflected and accelerated.

(d) Trajectories of typical reflected H^+ ions around shock

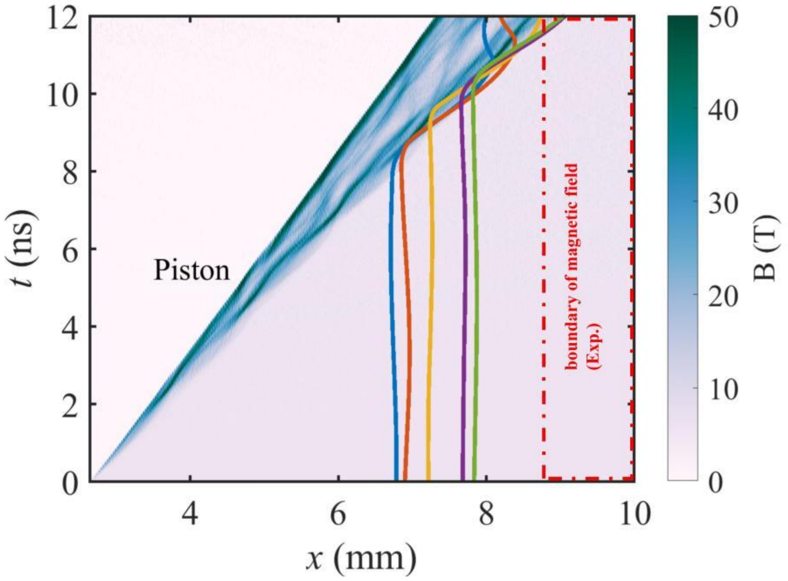

Fig. S10. Typical trajectories of accelerated ambient H^+ ions in 1-D PIC simulations. Five H^+ ions trajectories are illustrated with different colored lines. These ions, encounter the shock successively, are reflected and accelerated to the upstream region by the shock's electric field. The earlier reflected ions will enter the downstream due to the gyromotion in upstream *(57,63)*, while the remaining later reflected ions are still in the upstream region which may follow the shock, cross the boundary of magnetized ambient plasma with finite size, and move to the Faraday Cup to produce the quasi-monienergetic faster ions signals. 99.9% of reflected ions in our simulation experience single reflection and less than 0.1% ions can experience twice reflection.

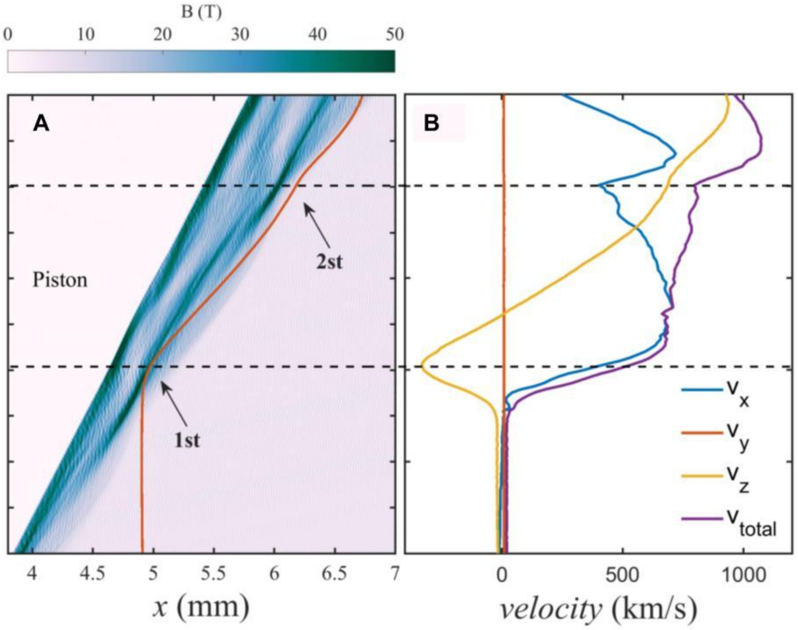

Fig. S11. Ions that experience twice reflections in 1-D PIC simulations with m_p/m_e=25. (**A**) A typical ambient H^+ ion trajectory (red) experience twice reflection, overlaid on the distribution of the magnetic field strength (color bar). (**B**) The temporal profile of the velocity gain of the reflected H^+ ion (v_x, v_y , v_z , and the total velocity v_{total}). Following the 1st reflection (SDA) and acceleration at about t_0+5ns, 2nd reflection (SSA) happens at about t_0+7ns accompanied with further ion acceleration. Less than 0.1% ions can experience twice reflection.

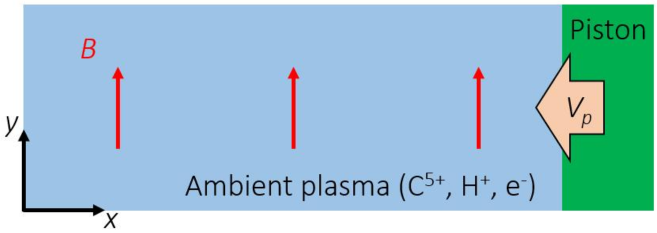

Fig. S12. 2-D PIC simulation setup. The simulation used a thin rectangular domain of L_x = 1020 c/ω_{pe} and L_y = 1 c/ω_{pe} in the x-y plane, with 40800 and 40 grids in the x and y directions, respectively. The piston flow drifts to the left and drives shock in the magnetized ambient plasma.

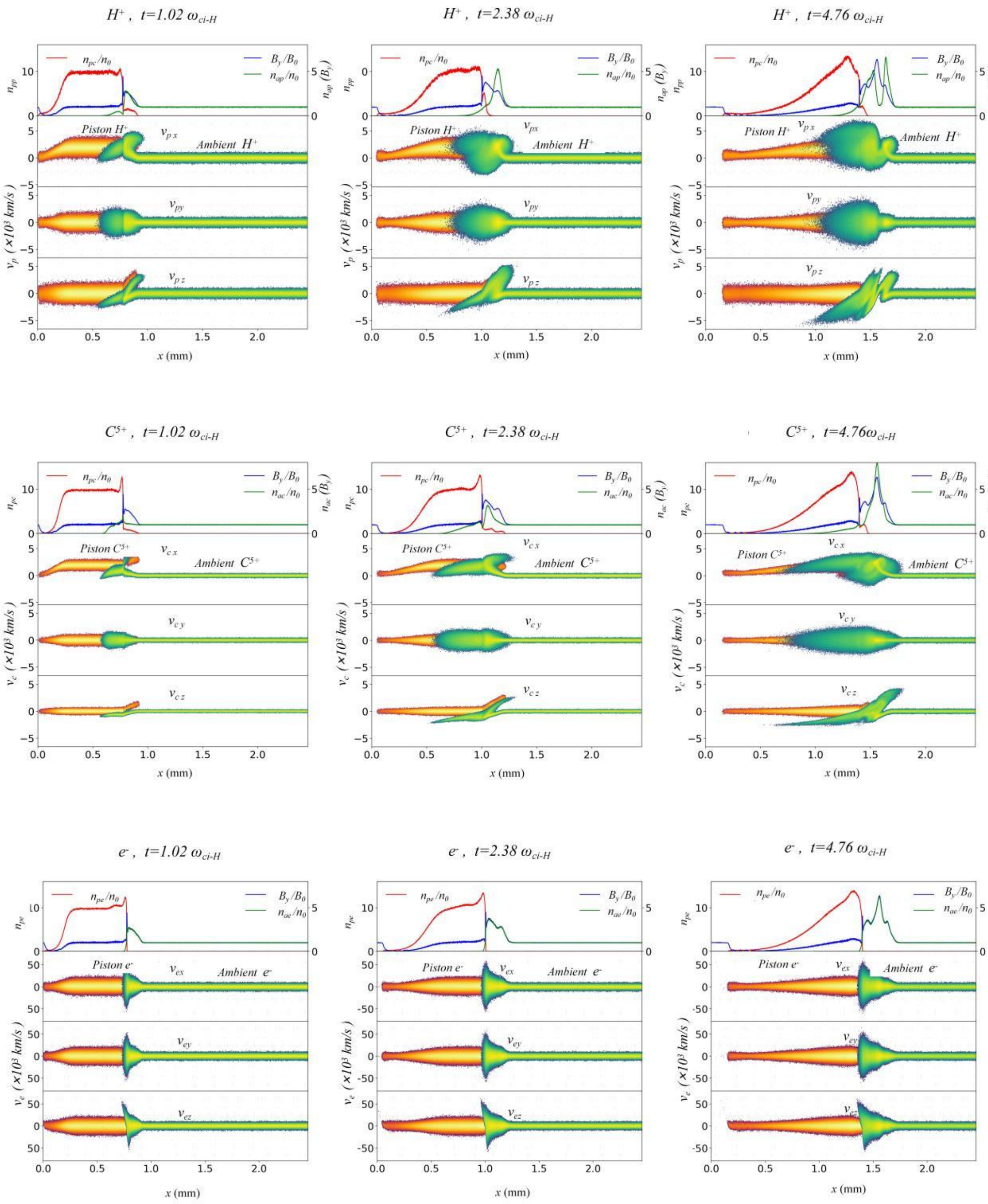

Fig. S13. 2-D PIC simulation results of ion dynamics during shock formation. The *v*-*x* phase space scatter plots of the H^+ ion (1st row), C^{5+} ion (2nd row), and electron (3rd row) are displayed associated with the magnetic fields B_y, ion and electron density profiles. At t=1.02 $\omega_{ci\text{-}H}^{-1}$ ($\omega_{ci\text{-}H}^{-1}$ is the H^+ ion gyroperiod), the upstream ions begin to be reflected. Then at t=2.38$\omega_{ci\text{-}H}^{-1}$ (close to the C^{5+} ion gyroperiod), both of the C^{5+} and H^+ ions form shock structures, and the shock of C^{5+}

moves slower behind of the H^+ shock. Finally, at t=4.76$\omega_{ci\text{-}H}^{-1}$, the shocks separate from the piston significantly.

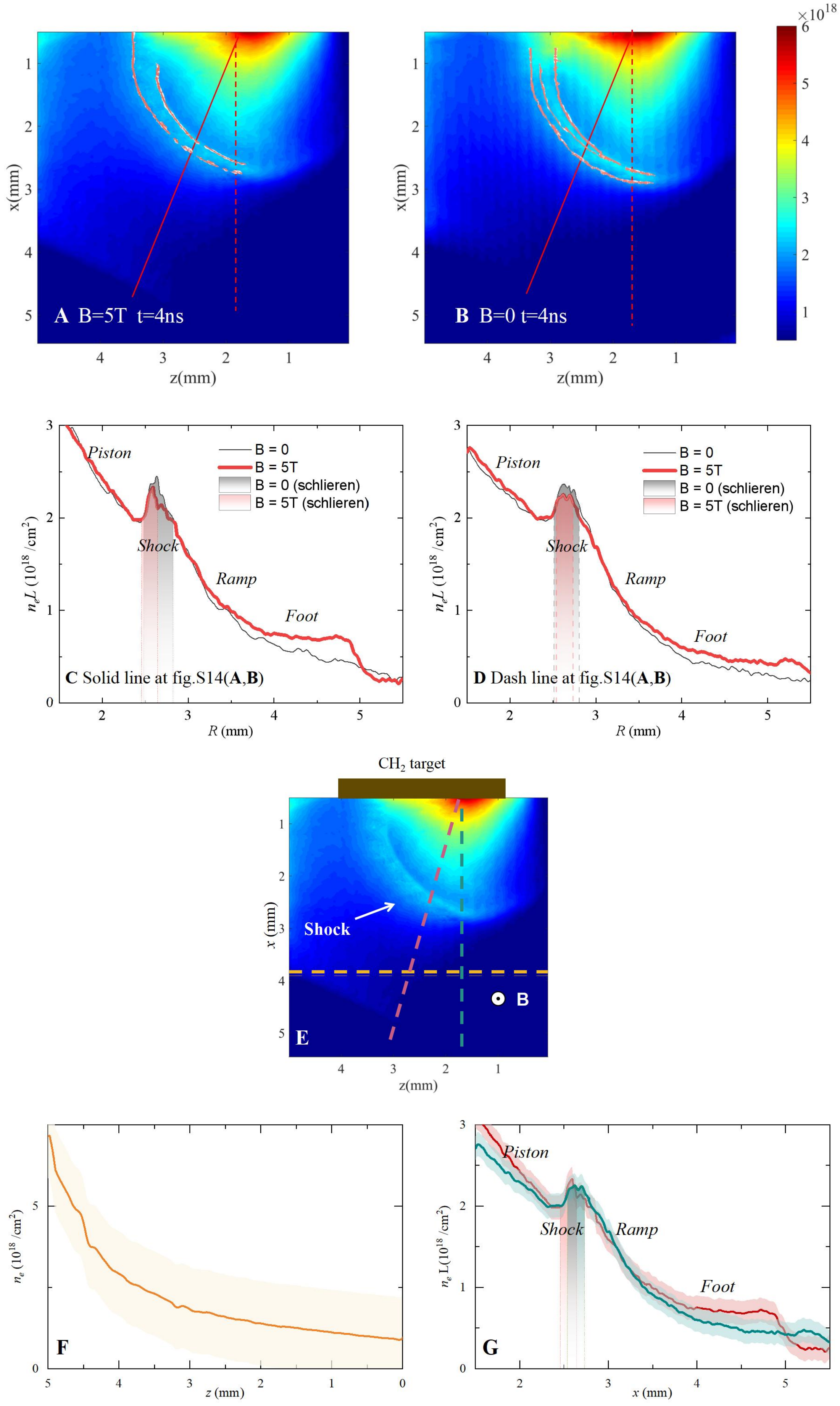
A B=5T t=4ns
B B=0 t=4ns
×10^18
x(mm)
z(mm)
C Solid line at fig.S14(A,B)
D Dash line at fig.S14(A,B)
B = 0
B = 5T
B = 0 (schlieren)
B = 5T (schlieren)
Piston
Shock
Ramp
Foot
n_eL (10^{18}/cm^2)
R (mm)
CH$_2$ target
Shock
B
E
F
n_e (10^{18}/cm^2)
z (mm)
G
n_e L(10^{18}/cm^2)
x (mm)

Fig. S14. The plasma imaging and density profile from optical diagnostics. The imaging of shock measured by optical interferometry and dark-field schlieren method (red contours) (line-integrated along y direction), taken at time t_0 + 4 ns formed in the ambient plasma for the cases of B=5T **(A)**, B=0 **(B)**. **(C, D)** Line-integrated electron density profile of shock taken along the solid line and dash line in S14 (**A**, **B**)**. (E)** same to fig1(b). **(F)** Electron density profile with error bar for the ambient plasma, taken at time t_0 + 4 ns along the yellow line in (c) at x=4 mm. **(G)** 1-D line-integrated electron density profile of shock taken along the red line and blue line in (**E**) with error bar.

REFERENCES AND NOTES

1. F. C. Jones, D. C. Ellison, The plasma physics of shock acceleration. *Space Sci. Rev.* **58**, 259–346 (1991).

2. R. Blandford, D. Eichler, Particle acceleration at astrophysical shocks A theory of cosmic ray origin. *Phys. Rep.* **154**, 1–75 (1987).

3. E. Fermi, On the origin of cosmic radiation. *Phys. Rev.* **75**, 1169–1174 (1949).

4. A. R. Bell, The acceleration of cosmic rays in shock front: I. *Mon. Not. R Astron. Soc.* **182**, 147–156 (1978).

5. A. R. Bell, The acceleration of cosmic rays in shock front: II. *Mon. Not. R Astron. Soc.* **182**, 443–455 (1978).

6. W. I. Axford, E. Lee, G. Skadron, The acceleration of cosmic rays by shock waves, in *Proceedings of the 15th International Cosmic Ray Conference (ICRC)* (IAEA, 1977), vol. 11, pp. 132.

7. G. F. Krymsky, A regular mechanism for the acceleration of charged particles on the front of a shock wave. *Sov. Phys. Dokl.* **22**, 327–328 (1977).

8. J. Matthews, A. Bell, K. Blundell, Particle acceleration in astrophysical jets. *New Astron. Rev.* **89**, 101543 (2020).

9. A. Marcowith, A. Bret, A. Bykov, M. E. Dieckman, L. O'C Drury, B. Lembège, M. Lemoine, G. Morlino, G. Murphy, G. Pelletier, I. Plotnikov, B. Reville, M. Riquelme, L. Sironi, A. Stockem Novo, The microphysics of collisionless shock waves. *Rep. Prog. Phys.* **79**, 046901 (2016).

10. A. Balogh, R. A. Treumann, Physics of Collisionless Shocks: Space Plasma Shock Waves (Springer, 2013).

11. R. Z. Sagdeev, Cooperative phenomena and shock waves in collisionless plasmas. *Rev. Plasma Phys.* **4**, 23–91 (1966).

12. A. S. Lipatov, G. P. Zank, H. L. Pauls, The acceleration of pickup ions at shock waves: Test particle-mesh simulations. *J. Geophys. Res.* **103**, 29679–29696 (1998).

13. R. B. Decker, Computer modeling of test particle acceleration at oblique shocks. *Space Sci. Rev.* **48**, 195–262 (1988).

14. M. Scholer, T. Terasawa, Ion reflection and dissipation at quasi-parallel collisionless shocks. *Geophys. Res. Lett.* **17**, 119–122 (1990).

15. M. Begelman, J. G. Kirk, Shock-drift particle acceleration in superluminal shocks: A model for hot spots in extragalactic radio sources. *Astrophys. J.* **353**, 66–80 (1990).

16. R. B. Decker, L. Vlahos, Shock drift acceleration in the presence of waves. *J. Geophys. Res.* **90**, 47–56 (1985).

17. S. V. Chalov, Shock drift acceleration of pickup protons at corotating interaction regions. *J. Geophys. Res.* **106**, 18667–18675 (2001).

18. D. Caprioli, A.-R. Pop, A. Spitkovsky, Simulations and theory of ion injection at non-relativistic collisionless shocks. *Astrophys. J. Lett.* **798**, L28 (2015).

19. T. Katsouleas, J. M. Dawson, Unlimited electron acceleration in laser-driven plasma waves. *Phys. Rev. Lett.* **51**, 392–395 (1983).

20. M. A. Lee, V. D. Shapiro, R. Z. Sagdeev, Pickup ion energization by shock surfing. *J. Geophys. Res.* **101**, 4777–4789 (1996).

21. G. P. Zank, H. L. Pauls, I. H. Cairns, G. M. Webb, Interstellar pickup ions and quasi-perpendicular shocks: Implications for the termination shock and interplanetary shocks. *J. Geophys. Res.* **101**, 457–477 (1996).

22. V. D. Shapiro, D. Üçer, Shock surfing acceleration. *Planet. Space Sci.* **51**, 665–680 (2003).

23. G. P. Zank, W. K. M. Rice, J. A. le Roux, I. H. Cairns, G. M. Webb, The "injection problem" for quasi-parallel shocks. *Phys. Plasmas* **8**, 4560–4576 (2001).

24. J. R. Jokipii, Rate of energy gain and maximum energy in diffusive shock acceleration. *Astrophys. J.* **313**, 842–846 (1987).

25. V. V. Lobzin, V. V. Krasnoselskikh, J.-M. Bosequed, J.-L. Pincon, S. J. Schwartz, M. Dunlop, Nonstationarity and reformation of high-Mach-number quasiperpendicular shocks: Cluster observations. *Geophys. Res. Lett.* **34**, L05107 (2007).

26. A. Johlander, S. J. Schwartz, A. Vaivads, Y. V. Khotyaintsev, I. Gingell, I. B. Peng, S. Markidis, P.-A. Lindqvist, R. E. Ergun, G. T. Marklund, F. Plaschke, W. Magnes, R. J. Strangeway, C. T. Russell, H. Wei, R. B. Torbert, W. R. Paterson, D. J. Gershman, J. C. Dorelli, L. A. Avanov, B. Lavraud, Y. Saito, B. L. Giles, C. J. Pollock, J. L. Burch, Rippled quasiperpendicular shock observed by the magnetospheric multiscale spacecraft. *Phys. Rev. Lett.* **117**, 165101 (2016).

27. Y. Kuramitsu, Y. Sakawa, T. Morita, C. D. Gregory, J. N. Waugh, S. Dono, H. Aoki, H. Tanji, M. Koenig, N. Woolsey, H. Takabe, Time evolution of collisionless shock in counterstreaming laser-produced plasmas. *Phys. Rev. Lett.* **106**, 175002 (2011).

28. L. Romagnani, S. V. Bulanov, M. Borghesi, P. Audebert, J. C. Gauthier, K. Löwenbrück, A. J. Mackinnon, P. Patel, G. Pretzler, T. Toncian, O. Willi, Observation of collisionless shocks in laser-plasma experiments. *Phys. Rev. Lett.* **101**, 025004 (2008).

29. H. Ahmed, M. E. Dieckmann, L. Romagnani, D. Doria, G. Sarri, M. Cerchez, E. Ianni, I. Kourakis, A. L. Giesecke, M. Notley, R. Prasad, K. Quinn, O. Willi, M. Borghesi, Time-resolved characterization of the formation of a collisionless shock. *Phys. Rev. Lett.* **110**, 205001 (2013).

30. N. L. Kugland, D. D. Ryutov, P.-Y. Chang, R. P. Drake, G. Fiksel, D. H. Froula, S. H. Glenzer, G. Gregori, M. Grosskopf, M. Koenig, Y. Kuramitsu, C. Kuranz, M. C. Levy, E. Liang, J. Meinecke, F. Miniati, T. Morita, A. Pelka, C. Plechaty, R. Presura, A. Ravasio, B. A. Remington, B. Reville, J. S. Ross, Y. Sakawa, A. Spitkovsky, H. Takabe, H.-S. Park, Self-organized electromagnetic field structures in laser-produced counter-streaming plasmas. *Nat. Phys.* **8**, 809–812 (2012).

31. W. Fox, G. Fiksel, A. Bhattacharjee, P.-Y. Chang, K. Germaschewski, S. X. Hu, P. M. Nilson, Filamentation instability of counterstreaming laser-driven plasmas. *Phys. Rev. Lett.* **111**, 225002 (2013).

32. C. M. Huntington, F. Fiuza, J. S. Ross, A. B. Zylstra, R. P. Drake, D. H. Froula, G. Gregori, N. L. Kugland, C. C. Kuranz, M. C. Levy, C. K. Li, J. Meinecke, T. Morita, R. Petrasso, C. Plechaty, B. A. Remington, D. D. Ryutov, Y. Sakawa, A. Spitkovsky, H. Takabe, H.-S. Park, Observation of magnetic field generation via the Weibel instability in interpenetrating plasma flows. *Nat. Phys.* **11**, 173–176 (2015).

33. J. S. Ross, D. P. Higginson, D. Ryutov, F. Fiuza, R. Hatarik, C. M. Huntington, D. H. Kalantar, A. Link, B. B. Pollock, B. A. Remington, H. G. Rinderknecht, G. F. Swadling, D. P. Turnbull, S. Weber, S. Wilks, D. H. Froula, M. J. Rosenberg, T. Morita, Y. Sakawa, H. Takabe, R. P. Drake, C. Kuranz, G. Gregori, J. Meinecke, M. C. Levy, M. Koenig, A. Spitkovsky, R. D. Petrasso, C. K. Li, H. Sio, B. Lahmann, A. B. Zylstra, H.-S. Park, Transition from collisional to collisionless regimes in interpenetrating plasma flows on the national ignition facility. *Phys. Rev. Lett.* **118**, 185003 (2017).

34. F. Fiuza, G. F. Swadling, A. Grassi, H. G. Rinderknecht, D. P. Higginson, D. D. Ryutov, C. Bruulsema, R. P. Drake, S. Funk, S. Glenzer, G. Gregori, C. K. Li, B. B. Pollock, B. A. Remington, J. S. Ross, W. Rozmus, Y. Sakawa, A. Spitkovsky, S. Wilks, H.-S. Park, Electron acceleration in laboratory-produced turbulent collisionless shocks. *Nat. Phys.* **16**, 916–920 (2020).

35. A. Rigby, F. Cruz, B. Albertazzi, R. Bamford, A. R. Bell, J. E. Cross, F. Fraschetti, P. Graham, Y. Hara, P. M. Kozlowski, Y. Kuramitsu, D. Q. Lamb, S. Lebedev, J. R. Marques, F. Miniati, T. Morita, M. Oliver, B. Reville, Y. Sakawa, S. Sarkar, C. Spindloe, R. Trines, P. Tzeferacos, L. O. Silva, R. Bingham, M. Koenig, G. Gregori, Electron acceleration by wave turbulence in a magnetized plasma. *Nat. Phys.* **14**, 475–479 (2018).

36. C. K. Li, V. T. Tikhonchuk, Q. Moreno, H. Sio, R. D. Petrasso, Collisionless shocks driven by supersonic plasma flows with self-generated magnetic fields. *Phys. Rev. Lett.* **123**, 055002 (2019).

37. C. Niemann, W. Gekelman, C. G. Constantin, E. T. Everson, D. B. Schaeffer, A. S. Bondarenko, S. E. Clark, D. Winske, S. Vincena, B. Van Compernolle, P. Pribyl, Observation of collisionless shocks in a large current-free laboratory plasma. *Geophys. Res. Lett.* **41**, 7413–7418 (2014).

38. D. B. Schaeffer, W. Fox, D. Haberberger, G. Fiksel, A. Bhattacharjee, Generation and evolution of high-mach-number laser-driven magnetized collisionless shocks in the laboratory. *Phys. Rev. Lett.* **119**, 025001 (2017).

39. D. B. Schaeffer, W. Fox, R. K. Follett, G. Fiksel, C. K. Li, J. Matteucci, A. Bhattacharjee, K. Germaschewski, Direct observations of particle dynamics in magnetized collisionless shock precursors in laser-produced plasmas. *Phys. Rev. Lett.* **122**, 245001 (2019).

40. W. Yao, A. Fazzini, S. N. Chen, K. Burdonov, P. Antici, J. Béard, S. Bolaños, A. Ciardi, R. Diab, E. D. Filippov, S. Kisyov, V. Lelasseux, M. Miceli, Q. Moreno, V. Nastasa, S. Orlando, S. Pikuz, D. C. Popescu, G. Revet, X. Ribeyre, E. d'Humières, J. Fuchs, Laboratory evidence for proton energization by collisionless shock surfing. *Nat. Phys.* **17**, 1177–1182 (2021).

41. Z. W. Yang, B. Lembège, Q. M. Lu, Impact of the rippling of a perpendicular shock front on ion dynamics. *J. Geophys. Res.* **117**, A07222 (2012).

42. Z. W. Yang, Q. M. Lu, B. Lembège, S. Wang, Shock front nonstationarity and ion acceleration in supercritical perpendicular shocks. *J. Geophys. Res.* **114**, A03111 (2009).

43. Z. Yang, Q. Lu, X. Gao, C. Huang, H. Yang, Y. Liu, H. Hu, D. Han, Magnetic ramp scale at supercritical perpendicular collisionless shocks: Full particle electromagnetic simulations. *Phys. Plasmas* **20**, 092116 (2013).

44. Y. F. Hao, Z. W. Yang, F. Guo, T. Z. Liu, X. L. Kong, L. C. Shan, D. J. Wu, Particle energization at a high Mach number perpendicular shock: 1D particle-in-cell simulations. *Astrophys. J.* **954**, 18 (2023).

45. J. Park, J. C. Workman, E. G. Blackman, C. Ren, R. Siller, Particle-in-cell simulations of particle energization from low Mach number fast mode shocks. *Phys. Plasmas.* **19**, 062904 (2012).

46. E. L. Lever, K. B. Quest, V. D. Shapiro, Shock surfing vs. shock drift acceleration. *Geophys. Res. Lett.* **28**, 7, 1367–1370 (2001).

47. M. Hoshino, N. Shimada, Nonthermal electrons at high mach number shocks: Electron shock surfing acceleration. *Astrophys. J.* **572**, 880–887 (2002).

48. H. Madanian, S. J. Schwartz, S. A. Fuselier, D. Burgess, D. L. Turner, L. J. Chen, M. I. Desai, M. J. Starkey, Direct evidence for magnetic reflection of heavy ions from high Mach number collisionless shocks. *Astrophys. J. Lett.* **915**, L19 (2021).

49. J. M. Broll, S. A. Fuselier, K. J. Trattner, S. J. Schwartz, J. L. Burch, B. L. Giles, B. J. Anderson, MMS observation of shock-reflected He^{++} at Earth's quasi-perpendicular bow shock. *Geophys. Res. Lett.* **45**, 49–55 (2018).

50. W. A. Livesey, C. T. Russell, C. F. Kennel, A comparison of specularly reflected gyrating ion orbits with observed shock foot thicknesses. *J. Geophys. Res.* **89**, 6824–6828 (1984).

51. E. L. M. Hanson, O. V. Agapitov, I. Y. Vasko, F. S. Mozer, V. Krasnoselskikh, S. D. Bale, L. Avanov, Y. Khotyaintsev, B. Giles, Shock drift acceleration of ions in an interplanetary shock observed by MMS. *Astrophys. J. Lett.* **891**, L26 (2020).

52. P. Hu, G.-Y. Hu, Y. L. Wang, H. B. Tang, J. Zheng, Pulsed magnetic field device for laser plasma experiments at Shenguang-II laser facility. *Rev. Sci. Instrum.* **91**, 014703 (2020).

53. N. Brenning, R. L. Merlino, D. Lundin, M. A. Raadu, U. Helmersson, Faster-than-Bohm cross-B electron transport in strongly pulsed plasmas. *Phys. Rev. Lett.* **103**, 225003 (2009).

54. D. B. Schaeffer, E. T. Everson, D. Winske, C. G. Constantin, A. S. Bondarenko, L. A. Morton, K. A. Flippo, D. S. Montgomery, S. A. Gaillard, C. Niemann, Generation of magnetized collisionless shocks by a novel, laser-driven magnetic piston. *Phys. Plasmas* **19**, 070702 (2012).

55. C. Niemann, W. Gekelman, C. G. Constantin, E. T. Everson, D. B. Schaeffer, S. E. Clark, D. Winske, A. B. Zylstra, P. Pribyl, S. K. P. Tripathi, D. Larson, S. H. Glenzer, A. S. Bondarenko, Dynamics of exploding plasmas in a large magnetized plasma. *Phys. Plasmas* **20**, 012108 (2013).

56. D. B. Schaeffer, W. Fox, D. Haberberger, G. Fiksel, R. K. Follett, High-Mach number laser-driven magnetized collisionless shocks. *Phys. Plasmas* **24**, 122702 (2017).

57. D. A. Tidman, N. A. Krall, *Shock Waves in Collisionless Plasmas* (Wiley-Interscience, 1971).

58. N. Sckopke, G. Paschmann, S. J. Bame, J. T. Gosling, C. T. Russell, Evolution of ion distributions across the nearly perpendicular bow shock: Specularly and non-specularly reflected-gyrating ions. *J. Geophys. Res.* **88**, 6121–6136 (1983).

59. J. A. Slavin, R. E. Holzer, Solar wind flow about the terrestrial planets 1. Modeling bow shock position and shape. *J. Geophys. Res.* **86**, 11401–11418 (1981).

60. M. G. Kivelson, C. T. Russell, *Introduction to Space Physics* (Cambridge Univ. Press, 1995).

61. M. Oka, L. B. Wilson III, T. D. Phan, A. J. Hull, T. Amano, M. Hoshino, M. R. Argall, O. Le Contel, O. Agapitov, D. J. Gershman, Y. V. Khotyaintsev, J. L. Burch, R. B. Torbert, C. Pollock, J. C. Dorelli, B. L. Giles, T. E. Moore, Y. Saito, L. A. Avanov, W. Paterson, R. E. Ergun, R. J. Strangeway, C. T. Russell, P. A. Lindqvist, Electron scattering by high-frequency whistler waves at Earth's Bow shock. *Astrophys. J. Lett.* **842**, L11 (2017).

62. D. B. Schaeffer, W. Fox, J. Matteucci, K. V. Lezhnin, A. Bhattacharjee, K. Germaschewski, Kinetic simulations of piston-driven collisionless shock formation in magnetized laboratory plasmas. *Phys. Plasmas* **27**, 042901 (2020).

63. A. S. Bondarenko, D. B. Schaeffer, E. T. Everson, S. E. Clark, B. R. Lee, C. G. Constantin, S. Vincena, B. Van Compernolle, S. K. P. Tripathi, D. Winske, C. Niemann, Collisionless momentum transfer in space and astrophysical explosions. *Nat. Phys.* **13**, 573–577 (2017).

64. F. Guo, J. Giacalone, The effect of large scale magnetic turbulence on the acceleration of electrons by perpendicular collisionless shocks. *Astrophys. J.* **715**, 406–411 (2010).

65. F. Guo, J. Giacalone, The acceleration of thermal protons at parallel collisionless shocks: Three-dimensional hybrid simulations. *Astrophys. J.* **773**, 158 (2013).

66. D. Haberberger, S. Tochitsky, F. Fiuza, C. Gong, R. A. Fonseca, L. O. Silva, W. B. Mori, C. Joshi, Collisionless shocks in laser-produced plasma generate monoenergetic high-energy proton beams. *Nat. Phys.* **8**, 95–99 (2011).

67. J. R. Rygg, F. H. Segun, C. K. Li, J. A. Frenje, M. J.-E. Manuel, R. D. Petrasso, R. Betti, J. A. Delettrez, O. V. Gotchev, J. P. Knauer, D. D. Meyerhofer, F. J. Marshall, C. Stoeckl, W. Theobald, Proton radiography of inertial fusion implosions. *Science* **319**, 1223–1225 (2008).

68. C. K. Li, F. H. Séguin, J. A. Frenje, J. R. Rygg, R. D. Petrasso, R. P. J. Town, O. L. Landen, J. P. Knauer, V. A. Smalyuk, Observation of the decay dynamics and instabilities of megagauss field structures in laser-produced plasmas. *Phys. Rev. Lett.* **99**, 015001 (2007).

69. G. Gregori, A. Ravasio, C. D. Murphy, K. Schaar, A. Baird, A. R. Bell, A. Benuzzi-Mounaix, R. Bingham, C. Constantin, R. P. Drake, M. Edwards, E. T. Everson, C. D. Gregory, Y. Kuramitsu, W. Lau, J. Mithen, C. Niemann, H.-S. Park, B. A. Remington, B. Reville, A. P. L. Robinson, D. D. Ryutov, Y. Sakawa, S. Yang, N. C. Woolsey, M. Koenig, F. Miniati, Generation of scaled protogalactic seed magnetic fields in laser-produced shock waves. *Nature* **481**, 7382, 480–483 (2012).

70. L. Gao, P. M. Nilson, I. V. Igumenshchev, M. G. Haines, D. H. Froula, R. Betti, D. D. Meyerhofer, Precision mapping of laser-driven magnetic fields and their evolution in high-energy-density plasmas. *Phys. Rev. Lett.* **114**, 215003 (2015).

71. L. Q. Shan, H. B. Cai, W. S. Zhang, Q. Tang, F. Zhang, Z. F. Song, B. Bi, F. J. Ge, J. B. Chen, D. X. Liu, W. W. Wang, Z. H. Yang, W. Qi, C. Tian, Z. Q. Yuan, B. Zhang, L. Yang, J. L. Jiao, B. Cui, W. M. Zhou, L. F. Cao, C. T. Zhou, Y. Q. Gu, B. H. Zhang, S. P. Zhu, X. T. He, Experimental evidence of kinetic effects in indirect-drive inertial confinement fusion hohlraums. *Phys. Rev. Lett.* **120**, 195001 (2018).

72. P. L. Pritchett, “Particle-in-cell simulation of plasmas—A tutorial” in *Space Plasma Simulation*, Lecture Notes in Physics 615 (Springer, 2003).

73. W. Fox, J. Matteucci, C. Moissard, D. B. Schaeffer, A. Bhattacharjee, K. Germaschewski, S. X. Hu, Kinetic simulation of magnetic field generation and collisionless shock formation in expanding laboratory plasmas. *Phys. Plasmas* **25**, 102106 (2018).

74. R. A. Treumann, Fundamentals of collisionless shocks for astrophysical application, 1. Non-relativistic shocks. *Astron. Astrophys. Rev.* **17**, 409–535 (2009).

75. R. A. Fonseca, L. O. Silva, F. S. Tsung, V. K. Decyk, W. Lu, C. Ren, W. B. Mori, S. Deng, S. Lee, T. Katsouleas, J. C. Adam, Osiris: A three-dimensional, fully relativistic particle in cell code for modeling plasma based accelerators, in *International Conference on Computational Science* (Springer, 2002), pp. 342–351.

76. J. D. Richardson, J. C. Kasper, C. Wang, J. W. Belcher, A. J. Lazarus, Cool heliosheath plasma and deceleration of the upstream solar wind at the termination shock. *Nature* **454**, 63–66 (2008).

77. D. Margarone, J. Krása, L. Giuffrida, A. Picciotto, L. Torrisi, T. Nowak, P. Musumeci, A. Velyhan, J. Prokůpek, L. Láska, T. Mocek, J. Ullschmied, B. Rus, Full characterization of laser-accelerated ion beams using Faraday cup, silicon carbide, and single-crystal diamond detectors. *J. Appl. Phys.* **109**, 103302 (2011).

78. Z. W. Yang, Q. M. Lu, S. Wang, The evolution of the electric field at a nonstationary perpendicular shock. *Phys. Plasmas* **16**, 124502 (2009).
